# Planar Cell Polarity Effector Fritz Interacts with Dishevelled and Has Multiple Functions in Regulating PCP

**DOI:** 10.1534/g3.116.038695

**Published:** 2017-03-02

**Authors:** Ying Wang, Victor F. Naturale, Paul N. Adler

**Affiliations:** Biology Department, University of Virginia, Charlottesville, Virginia 22903; Cell Biology Department, University of Virginia, Charlottesville, Virginia 22903

**Keywords:** *Drosophila*, Planar Polarity Effector, Planar Cell Polarity, *fritz*, *inturned*

## Abstract

The Planar cell Polarity Effector (PPE) genes *inturned*, *fuzzy*, and *fritz* are downstream components in the *frizzled/starry night* signaling pathway, and their function is instructed by upstream Planar Cell Polarity (PCP) core genes such as *frizzled* and *dishevelled*. PPE proteins accumulate asymmetrically in wing cells and function in a protein complex mediated by direct interactions between In and Frtz and In and Fy. How the PCP proteins instruct the accumulation of PPE protein is unknown. We found a likely direct interaction between Dishevelled and Fritz and Dishevelled and Fuzzy that could play a role in this. We previously found that mild overexpression of *frtz* rescued a weak *in* allele. To determine if this was due to extra Frtz stabilizing mutant In or due to Frtz being able to bypass the need for In we generate a precise deletion of the *inturned* gene (*in^PD^*). We found that mild overexpression of Fritz partially rescued *in^PD^*, indicating that *fritz* has In independent activity in PCP. Previous studies of PPE proteins used fixed tissues, and did not provide any insights into the dynamic properties of PPE proteins. We used CRISPR/Cas9 genome editing technology to edit the *fritz* gene to add a green fluorescent protein tag. *fritz^m^*^NeonGreen^ provides complete rescue activity and works well for *in vivo* imaging. Our data showed that Fritz is very dynamic in epidermal cells and preferentially distributed to discrete membrane subdomains (“puncta”). Surprisingly, we found it in stripes in developing bristles.

Many tissues in animals display Planar Cell Polarity (PCP). The coordinate alignment of bristles and cuticular hairs in insects, scales in fish, feathers in birds, and hairs in mammals are easily visualized examples of PCP (Jenny and Mlodzik 2006). PCP also regulates the development of internal organs and tissues. For example, stereocilia in the mammalian inner ear and ommatidia in the *Drosophila* eye show PCP in their uniform orientation (Zheng *et al.* 1995; Wolff and Rubin 1998; Dabdoub and Kelley 2005). Convergent extension, aspects of kidney development, and several other developmental processes are also controlled by PCP (Keller *et al.* 2000; Wallingford *et al.* 2000, 2002; Babayeva *et al.* 2013; Goggolidou 2014).

Over the past several decades the importance of the *frizzled* (*fz*)/*starry night* (*stan*) signaling pathway in controlling PCP in both vertebrates and invertebrates has been intensively studied (Zheng *et al.* 1995; Wallingford *et al.* 2000; Wang *et al.* 2005; Wang and Nathans 2007; Goodrich and Strutt 2011; Adler 2012; Goggolidou 2014). Early research on *Drosophila* identified several groups of genes including the upstream PCP core genes and the downstream Planar Polarity Effector (PPE) genes. This pathway has been most intensively studied on the fly wing, where in wild type each cell forms a single distally pointing hair (Wong and Adler 1993; Adler 2012). Mutations in the upstream genes result in most cells producing a hair with abnormal polarity. Rarely, cells form two or three hairs. Mutations in the downstream PPE genes result in similar abnormal hair polarity but notably many cells form more than one hair (usually two or three) (Wong and Adler 1993; Adler 2012). The protein products of all the genes in the pathway accumulate asymmetrically in epithelial cells and this is thought to play a key role in their function (Wong and Adler 1993; Usui *et al.* 1999; Axelrod 2001; Feiguin *et al.* 2001; Strutt 2001; Tree *et al.* 2002; Bastock *et al.* 2003; Jenny *et al.* 2003; Adler *et al.* 2004; Das *et al.* 2004; Collier *et al.* 2005; Strutt and Warrington 2008). The PPE group of genes has not been as well studied as the upstream genes. The group includes three genes: *inturned* (*in*), *fuzzy* (*fy*), and *fritz* (*frtz*). The asymmetric accumulation of these proteins is dependent on and instructed by the upstream PCP proteins (Adler *et al.* 2004; Strutt and Warrington 2008). In PCP mutants the PPE proteins do not preferentially accumulate on the proximal edge of wing cells but they retain some activity as the frequent multiple hair cells seen in PPE mutants are not seen in PCP mutants. The mechanism by which the PCP proteins instruct the accumulation of PPE proteins is unknown although in mammals the upstream Dishevelled (Dsh) protein has been shown to bind the Fuz PPE protein (*fuz* is the mammalian homolog of *fy*) (Zilber *et al.* 2013). We found this interaction to be conserved in *Drosophila* and that the Frtz PPE protein can also bind Dsh.

A variety of information has supported the idea that the three *Drosophila* PPE genes and proteins function together as a unit in fly PCP. This was first suggested due to the similarity of the mutant phenotypes for these genes and because double mutants for strong alleles of any of the PPE genes show a similar phenotype to each single mutant (Wong and Adler 1993; Adler *et al.* 2004; Collier *et al.* 2005). Further double mutants of weak alleles of any two PPE genes resulted in a synergistic interaction to produce a strong phenotype (Collier *et al.* 2005). It is also known that the accumulation of each of the PPE proteins is dependent on the other members of the group although there is some complexity to this interaction (Adler *et al.* 2004; Strutt and Warrington 2008; Wang *et al.* 2014). Loss-of-function mutations in any of these genes result in a decrease in the level of endogenous Frtz and In (Wang *et al.* 2014; Adler *et al.* 2004; Strutt and Warrington 2008). We also previously reported that moderate overexpression of *frtz* increased the accumulation of endogenous In while overexpression of *fy* decreased the accumulation of endogenous In (Wang *et al.* 2014). We suggested this was responsible for the ability of *frtz* overexpression to suppress and the ability of *fy* overexpression to enhance a temperature-sensitive hypomorphic allele of *in* (*in^II53^*) (Wang *et al.* 2014). However, we could not rule out the possibility that these genetic interactions were due to overexpressed *frtz* or *fy* being able to bypass the need for functional *in*. To determine if all of the phenotypic interactions could simply be explained by *frtz* or *fy* stabilizing or destabilizing mutant In we generated a precise deletion of the *in* gene, *in^PD^*, using the CRISPR/Cas9 genome editing technique. We found overexpression of *frtz* could partially rescue *in^PD^* and that the overexpression of *fy* could enhance *in^PD^* establishing that *frtz* and *fy* can function partly in PCP in an In-independent manner.

To study the Frtz protein in living cells when expressed at normal levels we used CRISPR/Cas9 to add a green fluorescent protein, mNeonGreen, to the carboxyl terminus of the *frtz* gene. This edited gene displayed normal *frtz* function. *In vivo* imaging of Frtz^mNeonGreen^ showed that Frtz accumulated asymmetrically in wing, thorax, abdominal, and arista cells. Using time-lapse *in vivo* imaging, we found Frtz was very dynamic and preferentially distributed to discrete membrane subdomains. Fluorescence recovery after photo-bleaching (FRAP) studies established that the dynamics of Frtz accumulation was similar in the several cell types studied. We also found that Frtz accumulated in stripes in bristles and the pattern was dependent on the actin cytoskeleton.

## Materials and Methods

### Fly genetics

All flies were raised at 25° unless otherwise stated. Mutant stocks were either obtained from the Bloomington Drosophila Stock Center at Indiana University, from the Vienna Drosophila Resource Center (VDRC), generated in our laboratory, or were generous gifts from J. Axelrod, D. Strutt, or T. Uemura. To direct transgene expression, we used the Gal4/UAS system (Brand and Perrimon 1993). *dsh-myc* stock was obtained from Bloomington Drosophila Stock Center at Indiana University (stock number 25385). We also generated transgenic flies that expressed myc-Frtz-GFP from the *ubiquitin* promoter/enhancer (*ubi-Myc-Frtz-GFP*) (Sekelsky *et al.* 1995; Wang *et al.* 2014). This transgene provided complete rescue of *frtz* phenotypic null alleles.

### Antibodies

Anti-Myc antibody was obtained from Cell Signaling Technology. Anti-GFP antibody was obtained from Molecular Probes. Alexa 488- and Alexa 568-conjugated secondary antibodies were purchased from Molecular Probes. Anti-Frtz antibody was generated in rats using a Frtz protein made in *E. coli* and His tagged to facilitate purification. The expressed protein was found in inclusion bodies and solubilized and we do not know if it is in a native conformation. It was not able to detect the endogenous protein. The anti-In monoclonal antibody was generated in our laboratory and described in a previous paper (Adler *et al.* 2004).

### Immunostaining

A standard paraformaldehyde fixation and staining procedure was used (Adler *et al.* 2004). Briefly, fly pupae were fixed in 4% paraformaldehyde in phosphate buffered saline (PBS) for 1–2 hr at 4°. After fixation, wings were dissected, rinsed, blocked, and then stained overnight at 4° with primary antibodies in PBS, 0.3% Triton X-100, and 10% goat-serum. Secondary antibodies were applied for 2–3 hr at room temperature.

### Isolation of flies carrying edited genes

In all injections to edit genes, two types of plasmids were injected into embryos: plasmids that drive expression of the gRNAs from the U6 promoter (U6:1 or U6:3 promoter) and homology-dependent repair templates. The details of the construction of these plasmids can be found in Supplemental Material, File S1. The DNA injection into embryos was performed at Rainbow Transgenic Flies, Inc.

#### in^PD^:

Both gRNA-expressing plasmids and homology-directed repair (HDR) templates were injected into *nos-cas9 attp40* embryos. The gRNAs were targeted to sequences located 5′ and 3′ to the *in* gene. We obtained 30 adults from this injection and these were crossed to *in*^IH56^/TM3 flies. Four out of the 30 G0 flies gave rise to *in* mutant G1 progeny, all of which were subsequently found to be the result of a precise deletion of the *in* gene (*in*^PD^). Stocks were established and confirmed by genomic DNA PCR followed by DNA sequencing of PCR products. One of the lines expressed Ds-Red and was due to homology-dependent repair. The other three lines did not express Ds-Red and were presumably due to nonhomologous end joining.

#### Ruby-in:

Both gRNA plasmid and HDR template were injected into *nos-cas9 attp40* (18055). The G1 progeny carrying the inserts were selected by Ds-Red fluorescence, stocks established, and confirmed by genomic DNA PCR followed by DNA sequencing of PCR products. The Ds-Red segment was removed by crossing to a hs-cre stock.

#### frtz-mNeonGreen:

There are seven exons in the *frtz* gene. The two gRNA constructs were designed to specifically cut in the 7th exon of *frtz*. The HDR template included a mNeonGreen sequence, a DS-Red sequence to screen for edit-containing flies, and two 1.5-kb homology arms flanking both ends of the gRNAs cuts and the *frtz* 7th exon (Figure 5A). Figure 5A shows the *frtz* gene map, the two gRNAs’ targeting sites, and the design of the HDR template. Both the gRNA plasmid and HDR template were injected into a *nos-cas9 attp2* (21104) stock. The G0 flies were crossed to *w*; *CyO/Gla* flies and the G1 progeny screened for Ds-Red fluorescence. Genomic DNA was extracted from adult flies with Ds-Red fluorescence and PCR done to validate edited genes. Fifty percent of G0 progeny gave rise to G1 progeny that displayed Ds-Red fluorescence and 20% of those were confirmed to have an insertion of the mNeonGreen sequence at the correct genomic location. The Ds-Red segment was removed by crossing to an hs-cre stock.

### Yeast-two-hybrid assays

For the yeast-two-hybrid assays, we used yeast strain AH109 and Matchmaker Two-Hybrid System 3 from Clontech. Briefly, full-length or truncated cDNA was subcloned into *pGADT7* or *pGBKT7* vector using specific restriction enzyme sites or Gibson assembly. To test the interaction of defined protein partners, *pGADT7* and *pGBKT7* plasmids containing one of the two protein partners were cotransformed into yeasts. Transformed yeasts were selected by growing on two-marker dropout medium (SD/-Leu/-Trp). These two markers select for the presence of both plasmids. Colonies from the previous dropout medium were transferred to a four-marker dropout medium (SD/-Ade/-His/-Leu/-Trp) with X-α-Gal. Only colonies growing on four-marker plates were considered as evidence for a positive interaction.

### Quick preparation of genomic DNA from Drosophila

The following buffers were used in the experiment:

Buffer A (stored at room temperature): 100 mM Tris-Cl (pH 7.5); 100 mM EDTA; 100 mM NaCl; 0.5% SDS.
Buffer B (stored at 4°): 200 ml potassium acetate (5 M); 500 ml lithium chloride (6 M)

About 30 anesthetized flies were collected in a 1.5-ml microcentrifuge tube and ground in 200 μl buffer A with a disposable tissue grinder. An additional 200 μl of buffer A was added (to a total volume of 400 μl) and grinding was continued until only cuticles remained. Samples were incubated at 65° for 30 min and 800 μl of buffer B was added to each sample and mixed well by inverting the tube multiple times, and then incubated on ice for at least 10 min and up to a few hours. Samples were centrifuged at 12,000 rpm for 15 min at room temperature. One milliliter of the supernatant was transferred into a new microcentrifuge and 600 μl of isopropanol was added to each sample and mixed well by inverting the tube several times. This was then centrifuged at 12,000 rpm for 15 min at room temperature. The supernatant was discarded. The pellet was washed with 70% ethanol, air-dried, and resuspended in 150 μl of Tris-EDTA buffer.

### Co-immunoprecipitation and western blotting

The following genotypes were used in these experiments:

w; ptc-Gal4 dsh-myc; UAS-fy-GFP
w; ptc-Gal4 dsh-myc;UAS-(myc)_6_-frtz

One hundred and fifty to two hundred wing discs of transgenic flies were dissected from third instar larvae and homogenized in prechilled lysis buffer containing protease inhibitors. The lysis buffer (and other reagents) came from a kit (Roche – 11 719 394 001) and contained 50 mM Tris HCl, pH 7.5; 150 mM NaCl; 1% Nonidet P40; 0.5% deoxycholate. The extract was then spun to remove cell debris. Protein A agarose beads (Roche) were added to the extract and incubated for at least 3 hr to reduce the background of nonspecific binding. The extract was spun again and 7–10 µl desired antibody was added to the supernatant and incubated for 3–4 hr. New protein A agarose beads were then added and the sample was incubated overnight. The beads were pelleted and washed. Protein was released from the beads and analyzed by standard western blot procedures.

### Imaging

Adult wings were mounted in Euparal and bright field images obtained using a Spot RT camera and a Zeiss Axiskop II microscope. In most cases a 40× Apochromatic objective was used (oil, 1.4 NA).

Most live imaging was done using a Zeiss 780 confocal microscope and a 63× or 40× oil immersion lens. One panel was obtained using a Zeiss 880 confocal microscope equipped with an Airy Scanner. Fixed and stained samples were imaged using a Zeiss 780 confocal microscope and a 63× or 40× oil immersion lens. Final panels were assembled using Adobe Illustrator or Microsoft PowerPoint. *Frtz^mNeonGreen^*, *ubi-myc-frtz-gfp*, *ubi-myc-venus-in*, and *ubi-myc-frtz-mcherry* white pupae were collected then aged at 25° for either 30 hr for imaging wing, thorax, and arista cells or for ∼42 hr for imaging abdominal cells. Aged pupae were placed on double-sided tape attached to microscope slides. The pupal cases were peeled back over the region to be imaged. Two silicon rubber spacers were used to build a chamber and one small drop of halocarbon oil was applied to the cover slip to create a good optical interface.

For FRAP experiments we routinely bleached a part of one or more cells that contained an edge where the fluorescent protein preferentially accumulated. Recovery was typically followed for from 5 to 10 min with images taken from every 30 sec to every 2 min. The age of the pupae examined depended on the tissue being examined and we used time points where the fluorescence was optimal. For example, on the wing we used pupae 28-30 hrs after white prepupae (awp).

### Data availability

Key fly lines described for the first time in this paper (*e.g.*, *frtz*^NeonGreen^) will be sent to the Drosophila Stock Center in Bloomington to insure wide availability to the community. Other stocks or additional data will be provided by the principal investigator (P.N.A.) upon request. Plasmids and aliquots of antibodies will also be provided upon request. Substantial data and methods information are provided in File S1.

## Results

### Reanalyzing Frtz structure

In the original characterization of the *frtz* gene and protein the SMART annotation tool (Schultz *et al.* 1998; Collier *et al.* 2005) identified a pair of tandem WD40 repeats from aa 296 to 375. We reanalyzed the Frtz protein structure using WD40-repeat protein Structure Predictor (WDSP), which was developed specifically to identify WD40 repeats and predict the secondary structures of WD40 domains (Wu *et al.* 2012; Wang *et al.* 2015). Six WD40 repeats were predicted in the protein using WDSP (Table S1A). Similarly, seven WD40 repeats were predicted in the human Frtz (hFrtz) protein, which is five more than previously suggested (Cui *et al.* 2013) (Table S1B). We previously established that the ability of Frtz to bind to In as assayed by the yeast-two-hybrid system was associated with a fragment that contained the previously identified WD40 domains (Wang *et al.* 2014). This fragment contained aa 296–aa 375 in Frtz. Since fragments containing the other putative WD40 domains did not give a positive reaction it appears that In cannot bind to a generic WD40 motif but rather to specific surfaces in WD40-4 and WD-5 of Frtz. We attempted to identify residues critical for the interaction of Frtz and In by using the analysis of the Frtz WD4 and WD-5 repeats from WDSP and CN3D. We mutated to alanine the two WDSP predicted hotspots on the top surface and 10 additional amino acid residues predicted by CN3D to be on the top, inner, or outer peripheral surfaces of the WD-4 and WD-5 repeats, except for A343 and A377 which were mutated to Glycine (Table S2). All 12 mutations were introduced into a single *Drosophila* Frtz WD40 fragment including the WD-4 and WD-5 repeats. Surprisingly, this highly mutated fragment still interacted strongly with In in the yeast-two-hybrid system. We conclude from this result that an alternative surface (*e.g.*, bottom) in WD-4 and -5 is likely responsible for mediating the binding of Frtz to In.

### Dsh interacts with Fy and Frtz

**Figure 1:**
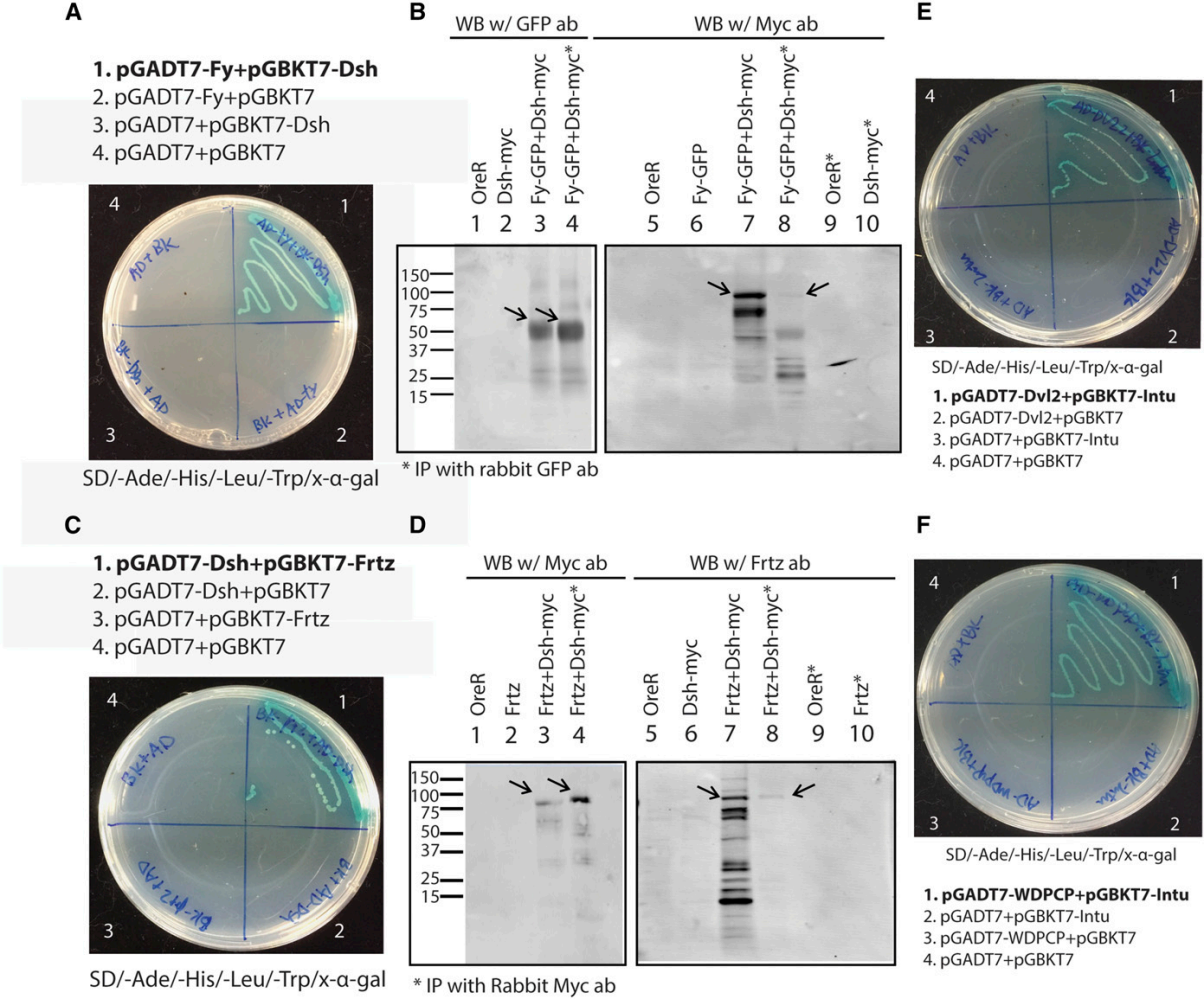
Protein interactions between PPE and PCP core proteins. (A and C) Yeast-two-hybrid plates for interaction between Fy and Dsh (A), Frtz and Dsh (C). The plates are 4 aa (Ade/-His/-Leu/-Trp) drop out plates containing x-α-gal. Only the colonies with interacting protein pairs grow and turn blue. (B and D) Co-immunoprecipitation results for Fy and Dsh (B) Frtz and Dsh (D), respectively. (B) The co-immunoprecipitation of Dsh and Fy. This experiment used *UAS-fy-GFP* driven by *ptc-Gal4* and *dsh-myc* transgenes (Bloomington Drosophila Stock Center stock number 25385). Wing disc samples were immunoprecipitated using rabbit GFP antibody then detected with mouse GFP and Myc antibody for western blotting. Arrows point to Fy-GFP and *dsh-myc* on the Western blots. Part D shows the co-immunoprecipitation of Dsh and Frtz. This experiment used *UAS-frtz* driven by *ptc-Gal4* and *dsh-myc* transgenes. Wing disc samples were immunoprecipitated using rabbit Myc antibody then detected with rat Frtz and mouse Myc antibody for western blotting. Arrows point to *dsh-myc* and Frtz on the Western blots. (E and F) Yeast-two-hybrid plates for interactions between Dvl2 and Intu (E) and WDPCP and Intu (F).

The In, Fy, and Frtz proteins accumulate on the proximal side of wing cells (Adler *et al.* 2004; Collier *et al.* 2005; Strutt and Warrington 2008; Wang *et al.* 2014) and function together in a protein complex (Wang *et al.* 2014). Early studies also showed the accumulation of PPE proteins Frtz and In on the proximal edge of wing cells was decreased in *fz* and *Vang* mutant clones (Adler *et al.* 2004; Strutt and Warrington 2008). Together it suggests one or more PCP core proteins are required for the asymmetric recruitments of PPE proteins or protein complex to the proximal edge. Using co-immunoprecipitation and the yeast-two-hybrid system, we found that the distally localized protein Dsh interacted with both Frtz and Fy (Figure 1, A–D and Table S3). Since an interaction between the mammalian Dsh homolog Dvl2 and the Fy homolog Fuz has been demonstrated (Zilber *et al.* 2013) we emphasized studying the interaction between Frtz and Dsh.

In order to determine what part of Frtz was responsible for the interaction with Dsh, several Frtz fragments were generated (Figure S1A), but only full-length Frtz interacted with Dsh, suggesting the interaction came from multiple locations all of which are required or that the protein as a whole is needed to fold into a conformation that allows the interaction with Dsh. In order to test these hypotheses, we generated a set of internally deleted *frtz1* genes where 300- or 450-bp fragments were deleted from the full-length cDNA. These alterations resulted in 100 or 150 aa deletions (Figure S1B). Each deletion was tested for an interaction with full-length Dsh in the yeast-two-hybrid assay. The yeast-two-hybrid data showed that Frtz proteins where aa 300–400, 400–500, 500–600, 600–700, or 800–951 were deleted still interacted with Dsh. In contrast Frtz proteins where aa 1–300 or 700–800 were deleted failed to interact with Dsh. The data argued that these two regions mediated the interaction between Frtz and Dsh and that both regions are required. WD40 domains 1–3 are found in the 1–300 fragment and no WD40 domains are present in aa 700–800. Thus, the WD40 domains are not sufficient for the interaction and other sequences are also required.

To determine if these interactions are conserved for their mammalian homologs, human *intu* (in homolog), *WDPCP* (*frtz* homolog), and *dvl2* (*dsh* homolog) were subcloned into yeast-two-hybrid vectors and protein interaction assays were performed (Table S3). We found Intu interacted with both Dvl2 and WDPCP (Figure 1, E and F). Thus, the interaction between In and Frtz detected in our studies on the fly proteins appears to be conserved. However, we did not see an interaction between In and Dsh, thus for these two proteins the fly model does not appear to mimic the human system. We also did not see an interaction between Dvl2 and WDPCP. Thus, the interaction between Frtz and Dsh that we detected with fly proteins may not be conserved in the human proteins. It remains possible that WDPCP interacts with another one of the Dvl isoforms, such as Dvl3.

### Generation of a precise deletion of in (*in*^PD^) using CRIPSR/Cas9

Previously, we described that the mild overexpression of Frtz almost completely suppressed the mutant phenotype of the temperature-sensitive hypomorphic allele *in*^II53^ and the overexpression of Fy enhanced the *in*^II53^ phenotype (Wang *et al.* 2014). More recently we found the mild overexpression of Frtz could suppress the strong *in*^IH56^ allele (Figure S2). This allele is due to a nonsense mutation as are most in alleles (Adler *et al.* 1994). Genes containing nonsense mutations can produce protein by read-through (Steneberg and Samakovlis 2001; Wang *et al.* 2001), and it seemed possible that the suppression of *in*^IH56^ by Frtz overexpression could be due to read-through and the resulting small amount of In protein being stabilized by the extra Frtz (Wang *et al.* 2014). Alternatively, it is possible that overexpressed Frtz can bypass the requirement for In. To distinguish between these models we needed to isolate an in mutation that we could be confident was a null allele.

**Figure 2:**
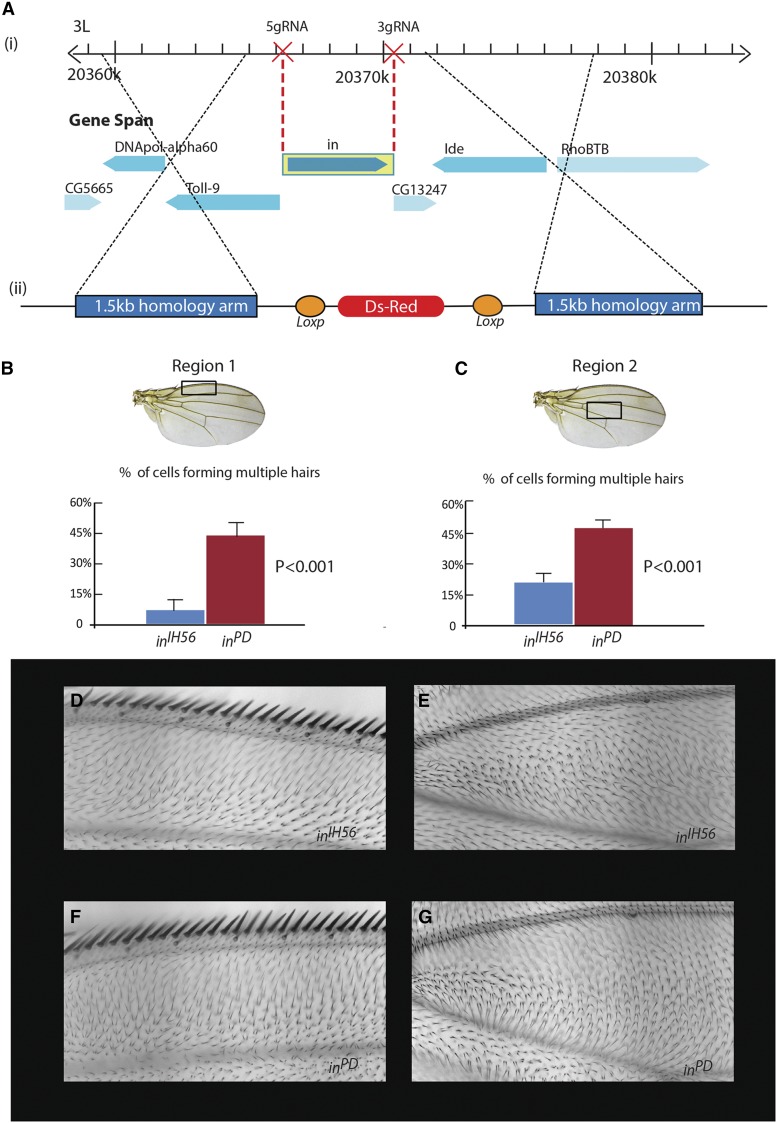
(A) The genomic region surrounding the in gene, the locations targeted by the gRNAs, and the homology fragment. (B) The phenotype of *in*^PD^ and *in*^IH56^ in the anterior region of the wing. Twenty wings were scored for each genotype to quantify the multiple hair cell phenotype. (C) The phenotype of *in*^PD^ and *in*^IH56^ in a central region of the wing. Twenty wings were scored for each genotype to quantify the multiple hair cell phenotype. (D) An image of the anterior region of the wing in *in*^IH56^. (E) An image of the central region of the wing in *in*^IH56^. (F) An image of the anterior region of the wing in *in*^PD^. (G) An image of the central region of the wing in *in*^PD^. Note the stronger phenotypes in *in*^PD^.

To generate an in null mutation, we used the CRISPR/Cas 9 genome editing technique (Gratz *et al.* 2013a,b; Ran *et al.* 2013). Figure 2A shows a genomic map of in and the location of the two gRNA targets and the HDR repair template. As described in *Materials and Methods* we obtained four precise deletions of the in gene. Since the *in* gene is completely deleted in *in*^PD^, no In protein can be produced in the mutant. In a number of wings region *in*^PD^ appeared to have a stronger mutant phenotype than *in*^IH56^ (Figure 2, B and C). We quantified the frequency of cells that formed multiple hairs in two such regions of the wing and found the *in*^PD^ wings had a stronger phenotype (Figure 2, D–G), which suggests the *in*^IH56^ nonsense mutation produces some protein by read-through. This is consistent with the hypothesis that the *frtz* suppression of in alleles is due to the stabilizing of mutant In by overexpressed Frtz. The hair polarity phenotype also appeared somewhat stronger in *in*^PD^, but this phenotype was not quantified.

**Figure 3:**
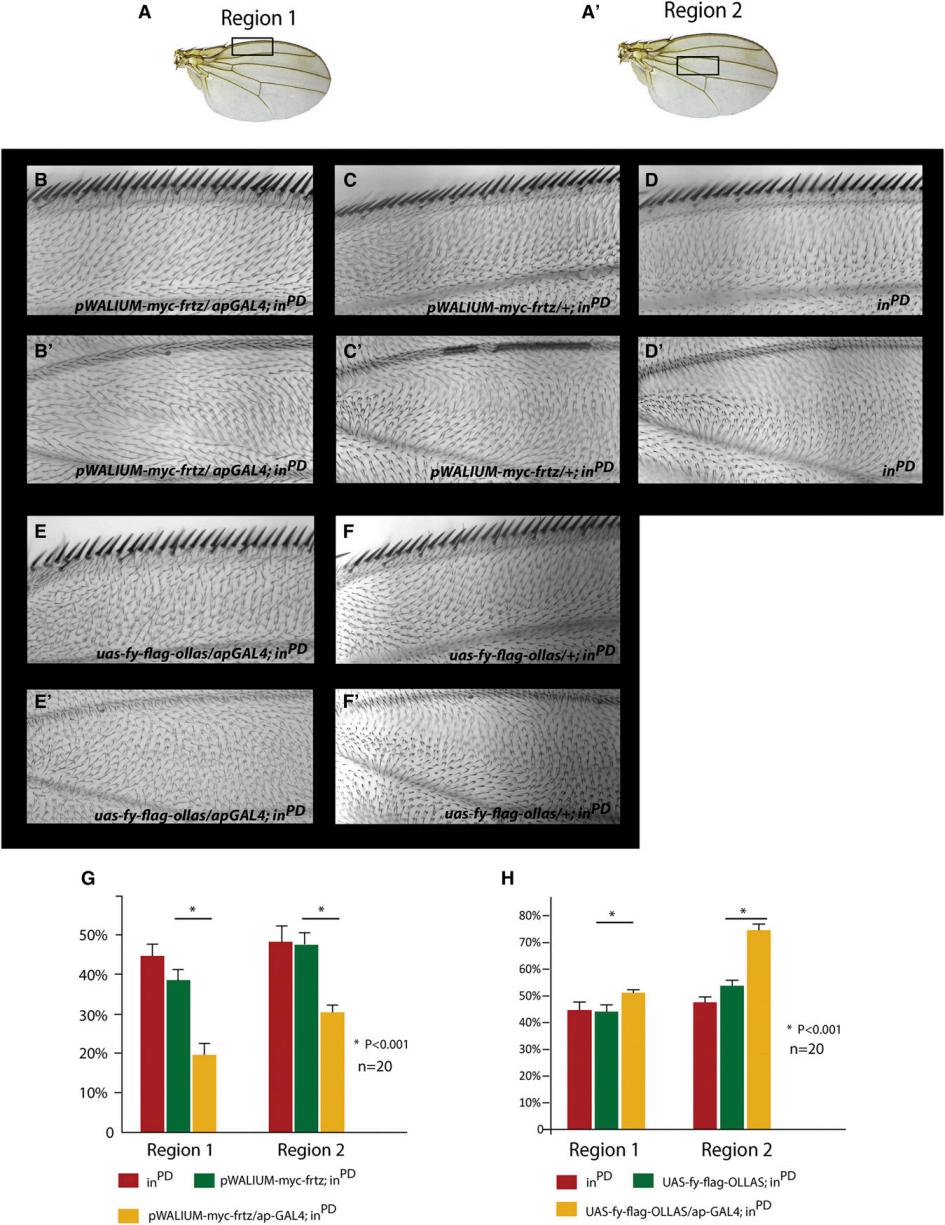
The mild overexpression of *myc-frtz* and *fy-flag-ollas* partially suppresses and enhances the phenotype of *in*^PD^, respectively. (A and A′) The regions of the wing scored are shown. (B and B′) Images of *ap > myc-frtz*; *in*^PD^ in the two wing regions. (C and C′) Images of *UAS-myc-frtz/+*; *in*^PD^ in the two wing regions. (D and D′) Images of *in*^PD^ in the two wing regions. (E and E′) Images of *ap > fy-flag-ollas; *in**^PD^ in the two wing regions. (F and F′) Images of *fy-flag-ollas/+; in*^PD^ in the two wing regions. (G) Quantitation of the multiple hair cell phenotype of the genotypes shown in B–D′. The numbers on the Y axis indicate the fraction of multiple hair cells. Significant differences are indicated with an asterisk. (H) Quantitation of the multiple hair cell phenotype of the genotypes shown in E–F′. The numbers on the Y axis indicate the fraction of multiple hair cells. Significant differences are indicated with an asterisk. *pWalium* is a *phiC31 UAS* germ line transformation vector (Perkins *et al.* 2015).

We then tested if mild overexpression of *frtz* could suppress the mutant phenotype of *in*^PD^, similarly to *in*^II53^and *in*^IH56^. *apterous-Gal4* (*ap-Gal4*) was used to drive the expression of *myc-frtz* in the dorsal layer of the wing. We analyzed the phenotype in two wing regions by observing hair polarity and by counting the number of multiple hair cells (Figure 3, B–D′). Both hair polarity and hair numbers in *in*^PD^ were weakly, but significantly rescued by overexpression of *frtz* (Figure 3G). We also observed a partial suppression of the abdominal bristle phenotype of *in*^PD^ with overexpression of *frtz* (Figure S3). We carried out an independent second test for the ability of *frtz* to suppress *in*^PD^ using a fusion protein-encoding gene (*UAS-frtz-cherry-pk*). The rationale for this experiment was that in the absence of In, Frtz would not localize to the proximal side of wing cells and that would hamper its ability to function in wing PCP. By fusing Frtz to Pk, which localizes proximally (Tree *et al.* 2002; Jenny *et al.* 2003), we hoped to drive Frtz to the proximal edge of wing cells independent of In. We established that the fusion protein had both Pk and In activity, as when expressed by *act-Gal4* it provided partial rescue of a *frtz* mutation (Figure S4, B and C). Further, when its expression was driven by the stronger *ptrc-Gal4* driver a typical *pk* gain of function was seen (Figure S4, E and F). Again the *in*^PD^ phenotype was weakly but significantly rescued by expressing the Frtz-Cherry-Pk fusion protein (Figure S4, G–I). However, the rescue was not better than that seen with *UAS-myc-frtz*. This could be due to the relatively poor accumulation of the fusion protein, perhaps due to folding problems. The partial rescue of the *in*^PD^ mutant phenotype in these experiments indicates that Frtz can function in part independently of In in PCP in *Drosophila*. Unlike the situation with *in*^II53^ and *in*^IH56^, the *in*^PD^ mutant phenotype was only weakly rescued consistent with Frtz having important In-dependent functions in PCP as well.

We previously found that the overexpression of Fy using *ap-Gal4* significantly enhanced the phenotype of *in*^II53^ and suggested this was due to Fy destabilizing the mutant In protein (Wang *et al.* 2014). Alternatively, overexpressed Fy could be impacting PCP in an In-independent manner. To distinguish between these hypotheses we tested if overexpressed Fy could enhance the *in*^PD^ phenotype. We found that it could. Overexpressing Fy using *apGal4* the percentage of cells forming multiple hairs was significantly increased in both the anterior and central domain of the wing (Figure 3, E–F′ and H). This established that Fy can impact PCP in an In-independent manner.

### Live imaging of transgenic Frtz protein

**Figure 4:**
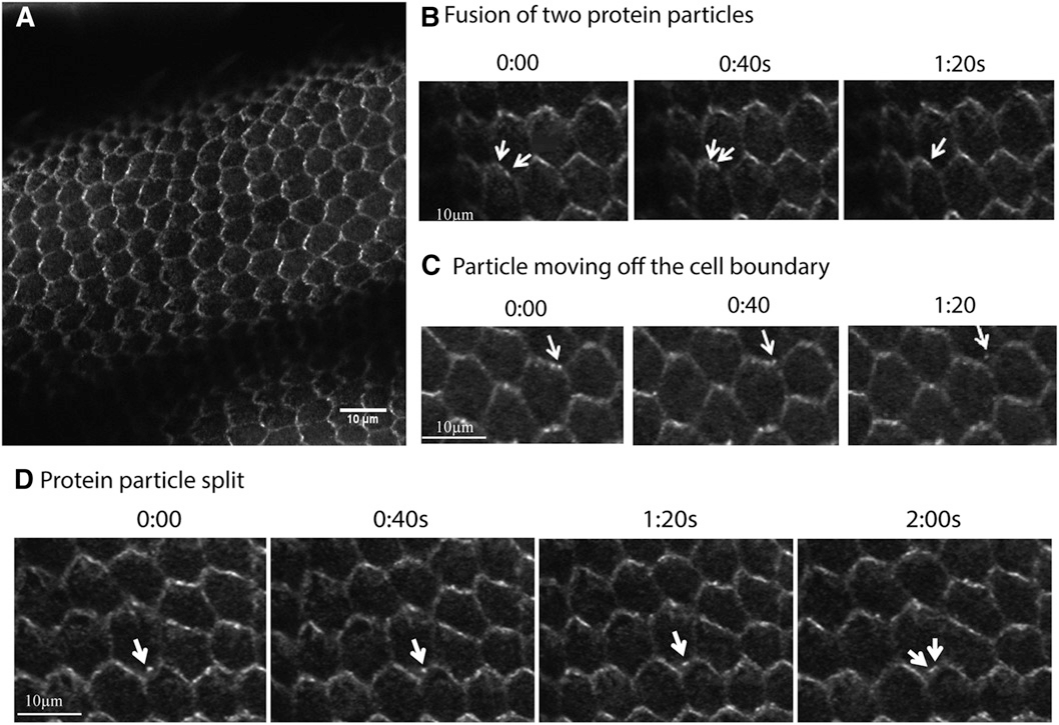
The *ubi-myc-frtz-GFP* transgene works well to show the asymmetric accumulation of the Frtz protein by *in vivo* imaging of pupae. (A) An image of a pupal wing. (B) A time series showing the fusion of a Frtz-GFP puncta. (C) A time series showing a puncta appearing to leave the membrane and move to the cytoplasm. (D) A time series showing a puncta that splits into two. The wings in these experiments were from 28- to 30-hr pupal wings.

The previous experiments on stained fixed wing tissues showed the Frtz protein asymmetrically localized at the proximal edge of wing cells (Strutt and Warrington 2008). However, those experiments did not provide any insight into the dynamic property of the Frtz protein. To understand how the Frtz protein functioned in live cells we first utilized flies that carried the *Ubi-myc-frtz-GFP* transgene. We observed the tagged protein in the wings of living pupae and observed that Frtz protein accumulated in the zig-zag pattern reported in experiments with fixed cells (Figure 4A) (Strutt and Warrington 2008). It was clear that the Frtz protein accumulated in discrete membrane subdomains (“puncta”) as is seen for the protein products of the upstream PCP genes (Strutt *et al.* 2011). The puncta were very dynamic with several types of movement observed including, but not limited to, puncta movement along the membrane, puncta fusion, puncta splitting into two, and puncta fusing with and leaving the membrane. Figure 4, B–D shows several examples of these.

With the goal of simultaneously imaging multiple PPE proteins *in vivo* we generated a set of constructs and transgenic flies where different colored fluorescent proteins were linked to PPE proteins (see File S1 for details) at the same PhiC31 landing site. Unfortunately, none of the transgenes tested resulted in as good imaging as the P insertions of *Ubi-myc-frtz*-GFP (see Figure 4). Using the attp1 landing site we were able to detect the zig-zag accumulation of Frtz and In (Figure S5, A and B and Table S4). The *in* transgene resulted in a higher number of puncta than we had seen previously for the endogenous protein by immunostaining experiments (compare Figure S5, B and C) (Adler *et al.* 2004). It was unclear if the high number of puncta was due to the protein behaving abnormally due to being tagged or to the ubiquitin enhancer/promoter resulting in an abnormal level of gene expression. In none of our experiments with Fy did we see asymmetric accumulation consistent with our previous attempts to detect tagged transgenic Fy using a transgene that contained the *fy* enhancer/promoter (Yan 2008).

All of the tagged transgenes provided full rescue of our strongest *frtz*, *in*, and *fy* alleles so the tagged proteins had activity and were expressed at a level that was compatible with *in vivo* function. However, the tagged PPE transgenes were less than ideal as the proteins were not expressed at the endogenous level and in some tissues, such as the developing adult abdominal epidermis, our ability to image the tagged protein was compromised by a higher level of expression in other cell types that were closely juxtaposed to the epidermis. With the development of CRISPR/Cas9 technology (Gratz *et al.* 2013a,b; Ran *et al.* 2013) the superior approach of tagging the endogenous gene became feasible and we took that approach.

**Figure 5:**
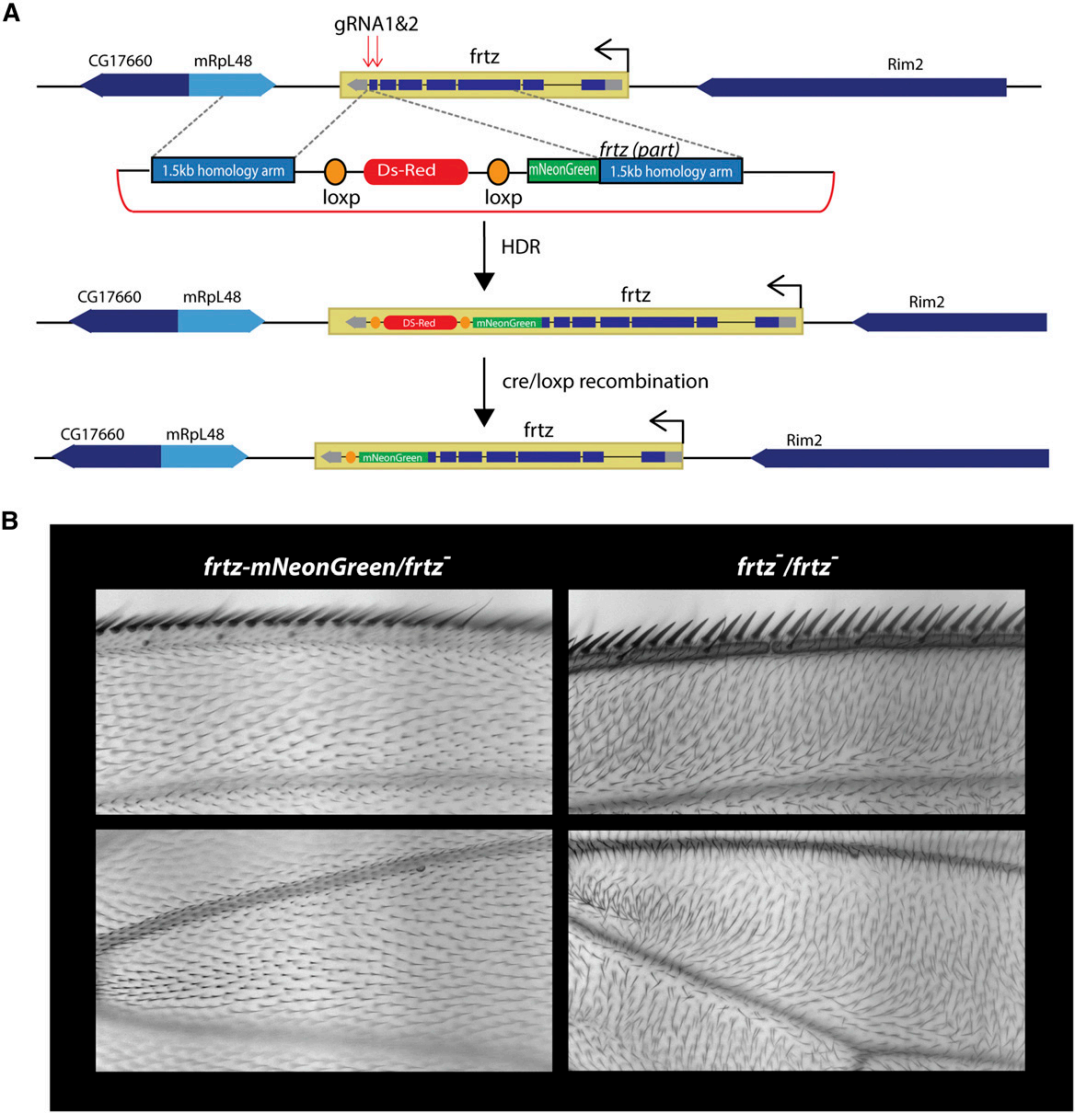
The CRISPR/Cas9-mediated editing of the endogenous *frtz* gene. (A) The genomic region around the *frtz* gene is shown along with the location of the gRNAs and the homology-dependent repair template. (B) Adult wings that show the *frtz^mNeonGreen^* edited gene is functional and provides complete rescue of a strong *frtz* allele.

**Figure 6:**
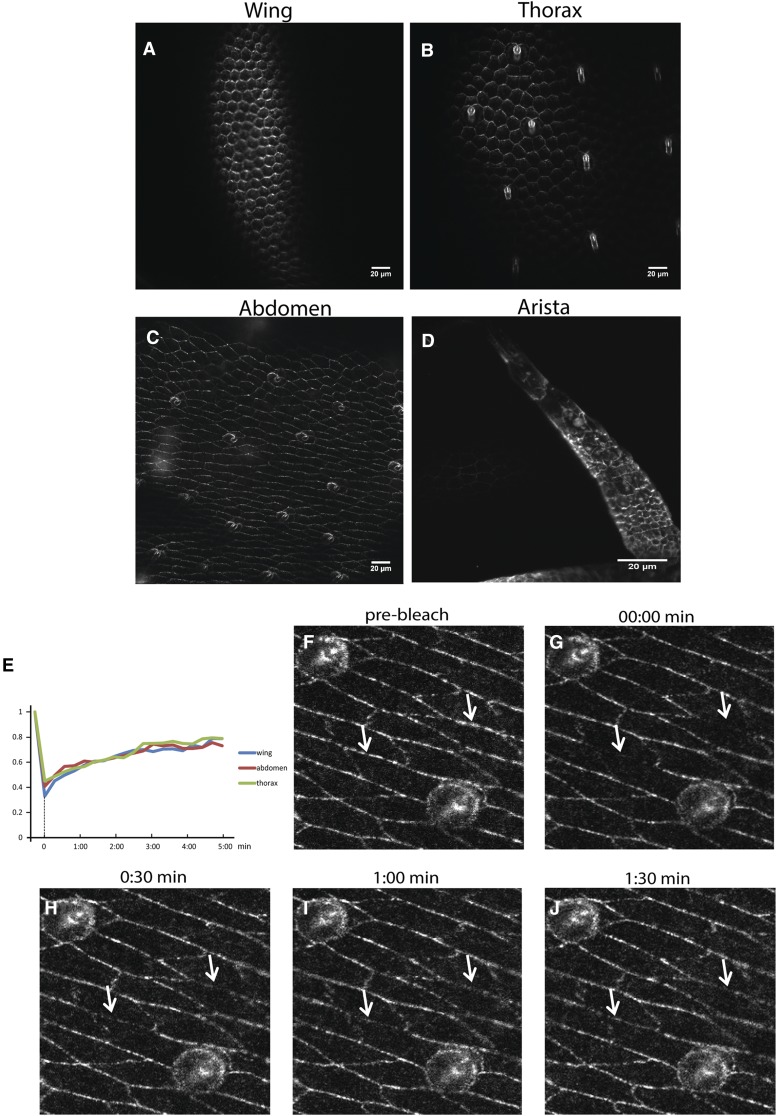
The edited *frtz^mNeonGreen^* gene is a very useful tool for *in vivo* imaging of the Frtz protein. (A) A 30-hr apf (after white prepupae formation) wing. (B) A 30-hr apf thorax. (C) A 43-hr apf abdomen. (D) A 28-hr apf antenna. (E) Quantitation of FRAP experiments on wing, thorax, and abdomen. (F–J) Time points from a FRAP experiment on the abdomen. The arrows in panels F–J indicate the location where cells were bleached and where one can follow recovery.

We used CRISPR/Cas9 to add a green fluorescent protein, mNeonGreen (Allele Biotechnology), to the carboxy terminus of the endogenous *frtz* gene (see *Materials and Methods* and Figure 5A). The edited *frtz^mNeonGreen^* gene completely rescued a *frtz* loss-of-function mutant and was wild type when homozygous suggesting the edited gene and fusion protein is fully functional (Figure 5B). We observed the asymmetric accumulation of Frtz*^mNeonGreen^* protein in both wing and thorax cells at 28–30 hr apf (after puparium formation) (Figure 6, A and B).

We also used the CRISPR/Cas9 to add the red fluorescent protein Ruby to the amino terminal end of *in*. The *ruby-in* gene provided rescue of *in* function and was wild type when homozygous. However, when we did *in vivo* imaging we observed a large number of puncta (Figure S5D), reminiscent of what we had observed with *ubi-venus-in*. This suggests the large number of puncta is a consequence of the fluorescent protein tag. Our best results in imaging Ruby-In came in experiments on the abdominal epithelial cells of 52 hr pupae and in these cells we could detect the asymmetric accumulation of Ruby-In, although there was substantial variation between individuals (see, for example, Figure S6, E and F). We did not examine this tissue in *ubi-venus-in* pupae. Further experiments will be required to find a location where a tag can be added without perturbing In dynamics in most cell types.

### Frtz protein functions and asymmetrically accumulates in the abdomen

**Figure 7:**
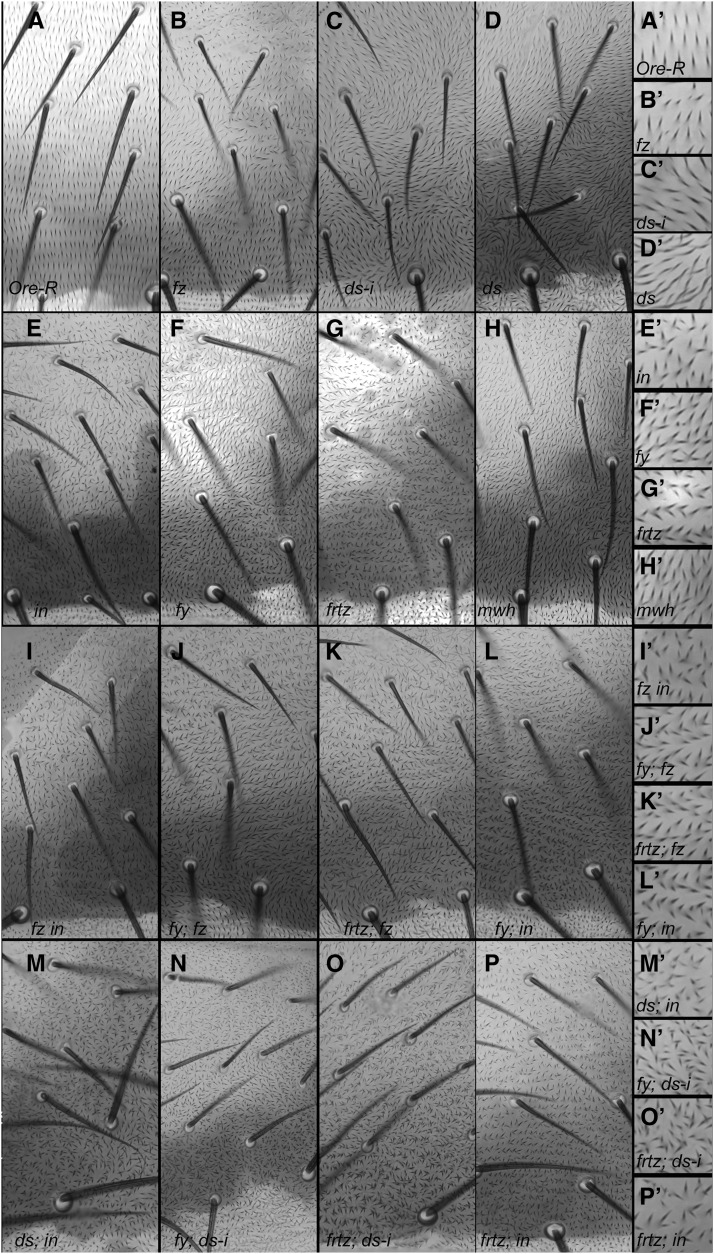
The phenotype of PPE mutants in the adult dorsal abdomen (tergite). All images are from a region just lateral to the midline of the 3rd abdominal segment. A higher magnification image from a region of each lower magnification is shown on the right and labeled with a ′. (A) Oregon-R. (B) *fz^P21^/fz^K21^*. (C) ptc > ds*-RNAi*. (D) *ds^UAO71^/ds^YAC41^*. (E) *in*^IH56^. (F) *fy^2^*. (G) *frtz^30^*. (H) *mwh^1^*. (I) *fz^1^ in^1^*. (J) *fy^2^*; *fz^1^*. (K) *frtz^30^*; *fz^1^*. (L) *fy^2^*; *in*^IH56^. (M) *ds^UAO71^/ds^YAC41^*; *in*^IH56^. (N) *fy^2^*; *ptc > ds-i*. (O) *frtz^30^*; *ptc > ds-I*. (P) *frtz^30^*; *in*^IH56^.

The adult abdomen stands out due to the phenotypic additivity of mutations in genes that are part of the *fz/stan* and *ds/ft* pathways (Casal *et al.* 2006; Donoughe and DiNardo 2011). It is known that PPE mutations alter bristle and hair polarity on the abdomen (Gubb and Garcia-Bellido 1982) but this aspect of PPE gene function has not been studied in depth. It is interesting that mutations in *fz/stan* PCP genes only result in grossly abnormal hair polarity over part of each abdominal segment (Casal *et al.* 2006) (Figure 7B), although in our hands the proteins appear to accumulate asymmetrically throughout the segment (Figure S6, A and B). This has also been seen by others in the embryonic, larval, and pupal abdomen (Price *et al.* 2006; Donoughe and DiNardo 2011; Sharp and Axelrod 2016). In contrast mutations in *frtz*, *in*, and *fy* produce strong hair phenotypes in all regions of abdominal segments where cells produce hairs (Figure 7, E–G). As in other body regions they consist of cells forming extra hairs and abnormal hair polarity. In double mutants between *fz* and any of the PPE mutations the hair phenotype resembled the PPE phenotype (Figure 7, I–K, I′–K′ *vs.* B, B′ and E–G, E′–G′). Thus in terms of the abdominal hair phenotype the PPE mutations are epistatic to *fz* mutations, as is the case for the wing hair phenotype (Wong and Adler 1993). Although we did not study it in as much detail we also observed that in double mutants between *ds* and any of the PPE mutants the hair phenotype resembled the PPE phenotype (Figure 7, M–O and M′–O′ compare with C–D and C′–D′). As is the case for the PPE mutant wing hair phenotype the phenotypes of PPE single and double mutants were similar (*e.g.*, Figure 7, E–G, E′–G′ *vs.* L, P and L′, P′). In contrast to the wing where the phenotype of *mwh* is stronger than the PPE mutants on the abdomen it was weaker than that of the PPE mutants (compare Figure 7, E–G *vs.* H, E′–G′ and H′). The dorsal abdominal bristle polarity of PPE mutants stands out due to bristles remaining aligned with their neighbors and a tendency to point to the midline (Gubb and Garcia-Bellido 1982). That differs from the more randomized bristle polarity of other PCP mutations. The bristle polarity phenotype of PPE/*fz* double mutants on the abdomen was intermediate between *fz* and PPE single mutants. Thus, in terms of the bristle polarity phenotype the PPE genes are not epistatic to the PCP genes. This is also true in the thorax. The basis for this is unclear.

Around 43 hr apf, we observed the asymmetric accumulation of Frtz protein in cells throughout the developing adult abdomen (Figure 6C). We were unable to detect Frtz^mNeonGreen^ in *dsh* mutants. This could be due to a lack of localization, reduced stability, or both. This is similar to what has been seen in the wing (Strutt and Warrington 2008). Similarly, we found that we could not detect Frtz^mNeonGreen^ in *in^PD^* homozygous flies either, consistent with the requirement for *in* for the accumulation of endogenous Frtz (Strutt and Warrington 2008). These data suggest that the PPE proteins function downstream of the PCP proteins in abdominal development for specifying hair number and polarity.

### Frtz protein functions and asymmetrically accumulates in the arista

Previous research in our laboratory showed the *fz/stan* signaling pathway regulates the development of *Drosophila* arista. Mutations in the PPE genes *in* and *fy* resulted in production of multipled and split laterals that were not evenly distributed along the central shaft (He and Adler 2002). We found that mutations in *frtz* also produced such a phenotype (Figure S7). We also previously observed that the Fz protein accumulated asymmetrically in the cells that form the central core of the arista (He and Adler 2002). Using the *frtz^mNeonGreen^* fly, we detected the asymmetric accumulation of Frtz in arista central core cells (Figure 6D) consistent with the asymmetric accumulation of Frtz being important for arista morphogenesis and for the pattern of Frtz protein being instructed by the upstream PCP proteins in this tissue.

### Stability of localized Frtz protein

In order to determine if there is a difference in the dynamic properties of the Frtz protein in different tissues, FRAP analysis was done on wing, thorax, and abdominal cells. In these experiments we routinely bleached a part of one or more cells over the region where Frtz^mNeonGreen^ preferentially accumulated. We then followed over time the reappearance of fluorescence in the region of preferential accumulation. After bleaching the cells, the fluorescence substantially recovered in all three tissues within 60 sec (Figure 6, E–J) and the estimated recovery half time is 70 sec. However, over the course of these experiments (5 min) we never detected a complete recovery of fluorescence. One explanation for this is that the cells contain both stable and less stable pools of localized Frtz protein. The stable protein pool would either not recover or recover slowly, while the unstable protein pool would recover rapidly. An alternative possibility is that the recovery came from Frtz^mNeonGreen^ protein dispersed in nonbleached regions of the cell and that there was not enough Frtz^mNeonGreen^ in this pool to provide for complete recovery.

### Accumulation of Frtz^mNeonGreen^ in hairs and sensory bristles

**Figure 8:**
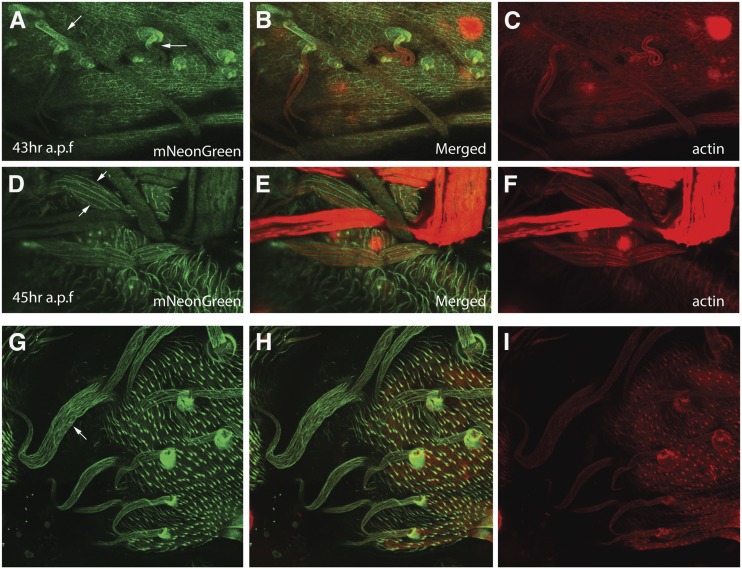
Frtz-NG accumulates in bristles and hairs. (A–C) Immunostained *frtz^mNeonGreen^* pupal head (43 hr after puparium formation). Frtz^mNeonGreen^ immunostaining in green and F-actin in red. The arrows point to the accumulation of Frtz^mNeonGreen^ in the proximal part of bristles. (D–F) Immunostained *frtz-NG* pupal head (45 hr after puparium formation). Frtz^mNeonGreen^ immunostaining in green and F-actin in red. The arrows point to the accumulation of Frtz^mNeonGreen^ in stripes down the bristles. From the merged image it is clear that the Frtz^mNeonGreen^ is in between large bundles of F-actin. (G–I) Immunostained *sn^3^ f^36a^*; *frtz^mNeonGreen^* pupal head (45 hr after puparium formation). Frtz^mNeonGreen^ immunostaining in green and F-actin in red. The arrow points to the accumulation of Frtz^mNeonGreen^ in disorganized stripes in the bristles. The actin staining image I shows the lack of large bundles of F-actin as expected for the *sn^3^ f^36a^* mutant.

Our live imaging of Frtz^mNeonGreen^ demonstrated the asymmetric accumulation of Frtz in various tissues. To our surprise, we also observed the accumulation of Frtz in *Drosophila* sensory bristles and hairs (Figure S6C). We also observed this in fixed preparations. Frtz^mNeonGreen^ started to accumulate in the proximal part of sensory bristles ∼43 hr apf (Figure 8A, arrows), and by ∼45 hr apf it reached its maximum intensity and accumulated throughout the entire bristles in a pattern of stripes parallel to the long axis of the bristle shaft (Figure 8D, arrows).

To further determine the localization of Frtz in bristles, we co-localized Frtz and F-actin in sensory bristles at 45 hr apf (Figure 8, D–F, arrows). Developing bristle shaft cells are characterized by a set of large bundles of F-actin (Tilney *et al.* 1995). The stripes of F-actin did not co-localize with the Frtz stripes, rather the Frtz stripes were localized to the region between the actin stripes. The Frtz stripes did not fill the space between the actin bundles and did not appear to contact the bundles. To determine if the bundles of F-actin found in the bristles were important for restricting the accumulation of Frtz we examined Frtz accumulation in an *sn^3^f^36a^* double mutant where the large actin bundles are missing (Figure 8, G–I) (Tilney *et al.* 1995). In the mutant bristles we still observed Frtz accumulation in stripes but they were no longer well organized and appeared increased in numbers indicating that in some way the actin bundles mediate the localization of Frtz.

In the experiments where we imaged Frtz^mNeonGreen^ in bristles we noticed strong staining in what appeared to be socket cells. In a 3-D reconstruction and from examining orthogonal views we observed that the high level of Frtz^mNeonGreen^ accumulation was likely in the socket cell where it is juxtaposed to the shaft cell (Figure S8 and File S2). The significance of this localization remains to be established.

## Discussion

### Interaction of Dsh with Fy and Frtz

The formation of the proximal and distal complexes of PCP proteins involves positive intercellular feedback and negative intracellular feedback. The proximal and distally localized cytoplasmic proteins are thought to be recruited both by a positive interaction with a similarly localized membrane protein (*e.g.*, between Fz and Dsh) (Chen *et al.* 2008; Strutt and Strutt 2008; Wu and Mlodzik 2008) and negative interactions between proximal and distally cytoplasmic proteins (*e.g.*, Pk and Dsh) (Tree *et al.* 2002; Amonlirdviman *et al.* 2005). Based on this example we expected that the proximal localization of PPE proteins would involve a positive recruitment by a proximally localized PCP protein and perhaps a negative interaction with a distally localized PCP protein. We detected interactions between both Fy and Dsh and Frtz and Dsh. An interaction between the mammalian homologs Fuz and Dvl was described previously (Zilber *et al.* 2013) and our experiments established that this interaction is conserved. We did not see such an interaction between the mammalian homologs of Frtz (WDPCP) and Dsh (Dvl2) so this interaction may not be conserved but such a negative result must be interpreted with caution. The interaction between the distally localized Dsh and the proximally localized Frtz and Fy is likely to be a negative one, perhaps analogous to the one seen between Dsh and Pk (Tree *et al.* 2002). That could result in incorrectly localized Frtz or Fy being degraded (Strutt *et al.* 2013; Cho *et al.* 2015).

Previous experiments established that the function of the upstream core PCP genes was essential for the proximal localization of In and Frtz to the proximal side of wing cells (Adler *et al.* 2004; Strutt and Warrington 2008). Based on those observations it seemed likely that one or more of the PPE proteins would bind to one or more of the proximally localized PCP proteins. We did not obtain convincing and reproducible evidence for such an interaction. That does not mean that such an interaction does not take place. For example, it is possible that the recruitment of one of the PPE proteins such as Frtz or In to the proximal side of wing cells could require an interaction with both Pk and Vang, an interaction with a modified protein or an interaction with an as yet unidentified proximal protein. It could also be weak enough for us not to be able to detect it. Our experiments with the Pk-Cherry-Frtz fusion protein establish that Pk and Frtz can function in close proximity.

### Not all PPE gene function requires the presence of all three members of the group

Most, but not all previous data on the PPE genes in *Drosophila* suggested that these genes and proteins functioned as a unit in PCP and each were required for the function of the others. In this paper we found that the overexpression of *frtz* could partially suppress and the overexpression of *fy* could weakly enhance the phenotype of a fly that completely lacked the in gene. Thus, in these experiments both Frtz and Fy were able to influence PCP in an In independent manner. Since the ability to influence the planar cell polarity phenotype required overexpression it is not clear that in a wild-type fly *frtz* and/or *fy* function in an *in*-independent manner but our results indicate the potential is there. This is consistent with previous results where we found the PCP gain-of-function phenotype that resulted from the overexpression of *frtz* or *frtz-GFP* was not eliminated by a lack of *in* function, but those experiments utilized an *in* nonsense mutation and a gain-of-function *frtz* phenotype (Wang *et al.* 2014). That both Frtz and Fy could impact PCP in the absence of any In suggests that its principal function might be to mediate the accumulation and localization of these proteins. When overexpressed enough Frtz and Fy might be present to allow a partial bypass of that function.

### Frtz and the actin cytoskeleton

In studying the expression and localization of Frtz using the *frtz^mNeongreen^* gene we discovered that Frtz accumulated in stripes along the proximal/distal axis of sensory bristles in the space between the large actin bundles found in bristles. In the absence of the F-actin bundles this pattern became disorganized and there appeared to be an increased number of Frtz stripes. This indicates that the patterning of the Frtz stripes is regulated by the actin cytoskeleton, although since the two did not overlap the regulation seems likely to be indirect. We previously found that the overexpression of *frtz* resulted in a delay in the activation of the actin cytoskeleton that drives wing hair morphogenesis (Wang *et al.* 2014). Further, the morphology of the actin cytoskeleton and the resulting hair were quite abnormal. Taken together it suggests that the actin cytoskeleton and Frtz mutually interact and regulate each other’s localization and activity. If and how this relates to the function of Frtz in PCP will require further research. It is interesting to note that in vertebrates the *WDPCP* mutant phenotype differs from that of *intu* and *fuz* (Kim *et al.* 2010; Cui *et al.* 2013) and that *WDPCP* mutants appear to have an actin cytoskeleton phenotype (Cui *et al.* 2013).

### PPE genes and PCP in the abdomen

We found that mutations in *Drosophila* PPE genes produced a strong multiple hair phenotype and a hair polarity phenotype in all regions of the dorsal abdomen. This differs from that seen with mutations in upstream *fz/stan* pathway genes where a strong hair polarity phenotype is only seen in part of each segment (Casal *et al.* 2006), even though PCP proteins accumulate asymmetrically in cells throughout segments. The *ds/ft* pathway also plays a key role in abdomen PCP, and double mutants where both pathways are inactivated show an additive phenotype with a strong phenotype in all regions (Casal *et al.* 2006). This raised the possibility that the PPE genes functioned downstream of both pathways in the abdomen and that the region-specific phenotypes of *fz/stan* and *ds/ft* could be due to two upstream pathways being dominant with regard to instructing the accumulation of PPE proteins in different regions of the segment. Consistent with the PPE genes functioning downstream of *fz/stan* in the abdomen, double mutants of *fz* with either *frtz*, *fy*, or in resembled the PPE hair phenotype. We found that both In and Frtz accumulated asymmetrically in abdominal cells throughout each segment consistent with that being important for their function. However, this asymmetric accumulation was lost throughout each segment in a *dsh*^1^ mutant background. Thus, having a functional *ds/ft* pathway is insufficient to promote asymmetric accumulation of PPE proteins in any part of the segment. It is worth noting that in the abdomen the PPE proteins retain substantial function even when not properly localized as the strong PPE multiple hair phenotype is not seen in a *dsh*^1^ mutant. This mirrors what is seen in the wing (Wong and Adler 1993; Adler *et al.* 2004).

### Two pools of Frtz protein within the cells

We analyzed the dynamics of Frtz in several body regions using FRAP on epithelial cells that expressed the edited *frtz^mNeonGreen^* gene. Similar results were obtained in all body regions. We found the recovery of most of the fluorescence was relatively fast (∼70 sec); however, the recovery did not go to completion over the time period (∼5–10 min) followed. The recovery was limited to ∼80% of the starting fluorescence. This could be due to two pools of Frtz protein within cells that vary in stability. Alternatively, it could be due to a lack of sufficient unbleached protein in the cell to allow complete recovery of fluorescence. We observed a greater fraction of recovery than was seen by Strutt *et al.* (2011) in their FRAP analysis on the PCP core proteins Fz and Stan. These two proteins are transmembrane proteins and that might be part of the reason for a larger fraction of stable and nonrecovering protein.

### Tagged endogenous genes *vs.* transgenes

The ability to edit the endogenous *frtz* gene provided us with a tool that was clearly superior to our previous generation *Ubi-myc-frtz-GFP* transgenes as we could be confident that the former was providing normal levels and developmental expression pattern. It also avoided the sometimes confounding effects of the artificial promoter/enhancer that resulted in *myc-frtz-GFP* being abnormally expressed in tissues that were closely juxtaposed to the epidermis, which hampered imaging. However, the *Ubi-myc-frtz-GFP* transgenes remain quite useful as they provide function and a tagged protein that is encoded at other locations in the genome (*e.g.*, 3rd chromosome). The flexibility that this provides is advantageous for some experiments that involve complicated genetics.

The ability to precisely delete the in gene using CRISPR/Cas9 allowed us to determine that small amounts of In protein activity were confounding some of our gene interaction experiments. In our case our nonsense allele of in proved not to be a “null” allele. It has long been true in some systems (*e.g.*, bacteria and yeast) that single gene deletions were easy to generate, and that is now the case generally and the advantages of such alleles are obvious.

## Supplementary Material

Supplemental material is available online at www.g3journal.org/lookup/suppl/doi:10.1534/g3.116.038695/-/DC1.
