# Supplementary figures and images for: Planar Cell Polarity Effector Fritz Interacts with Dishevelled and Has Multiple Functions in Regulating PCP
Source: G3 (Bethesda). 2017 Mar 2;7(4):1323–37. doi: 10.1534/g3.116.038695 (PMC5386880; doi:10.1534/g3.116.038695)

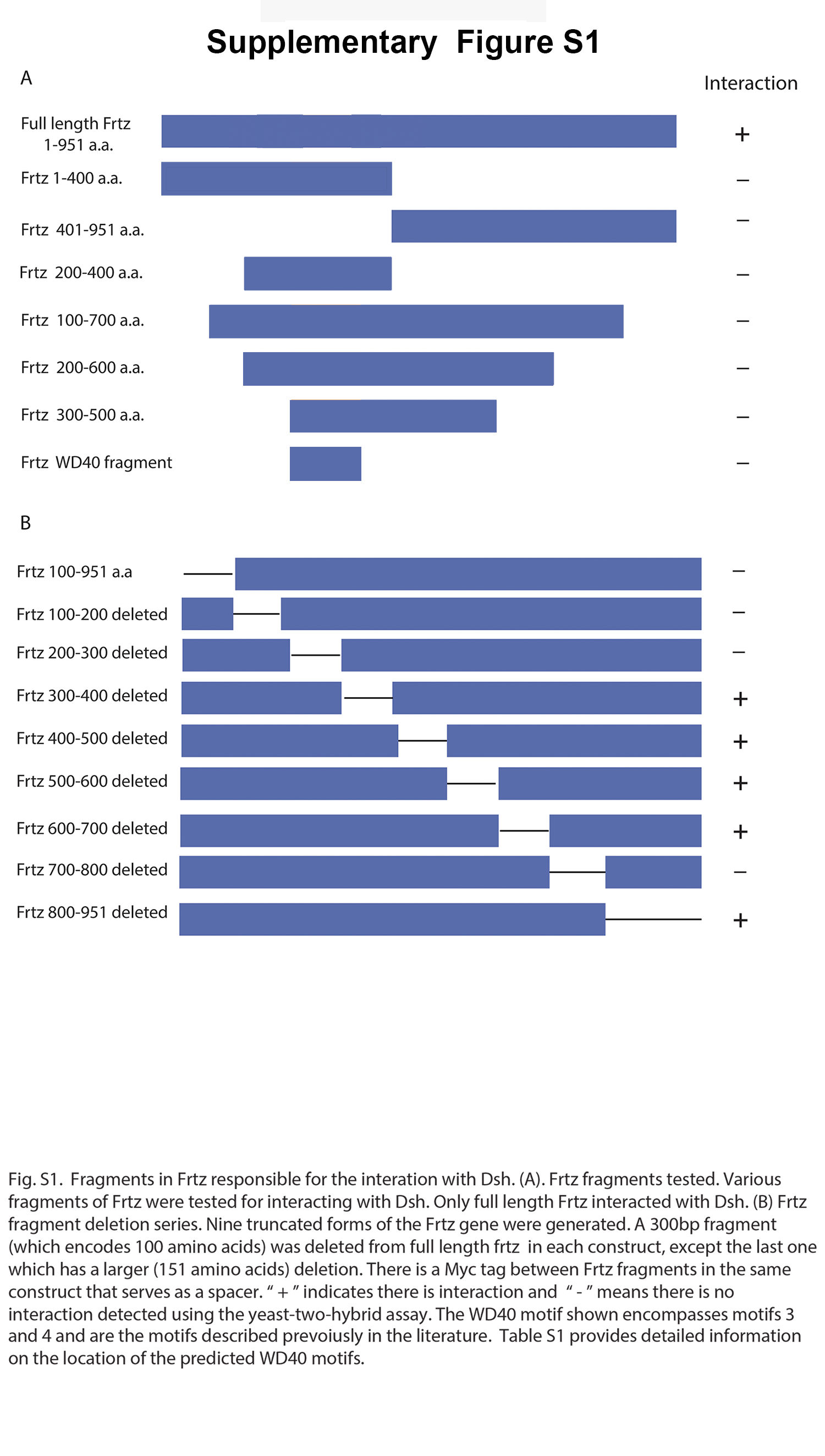

Supplement: Supplementary file 1 [file 1323FigureS1.tif]

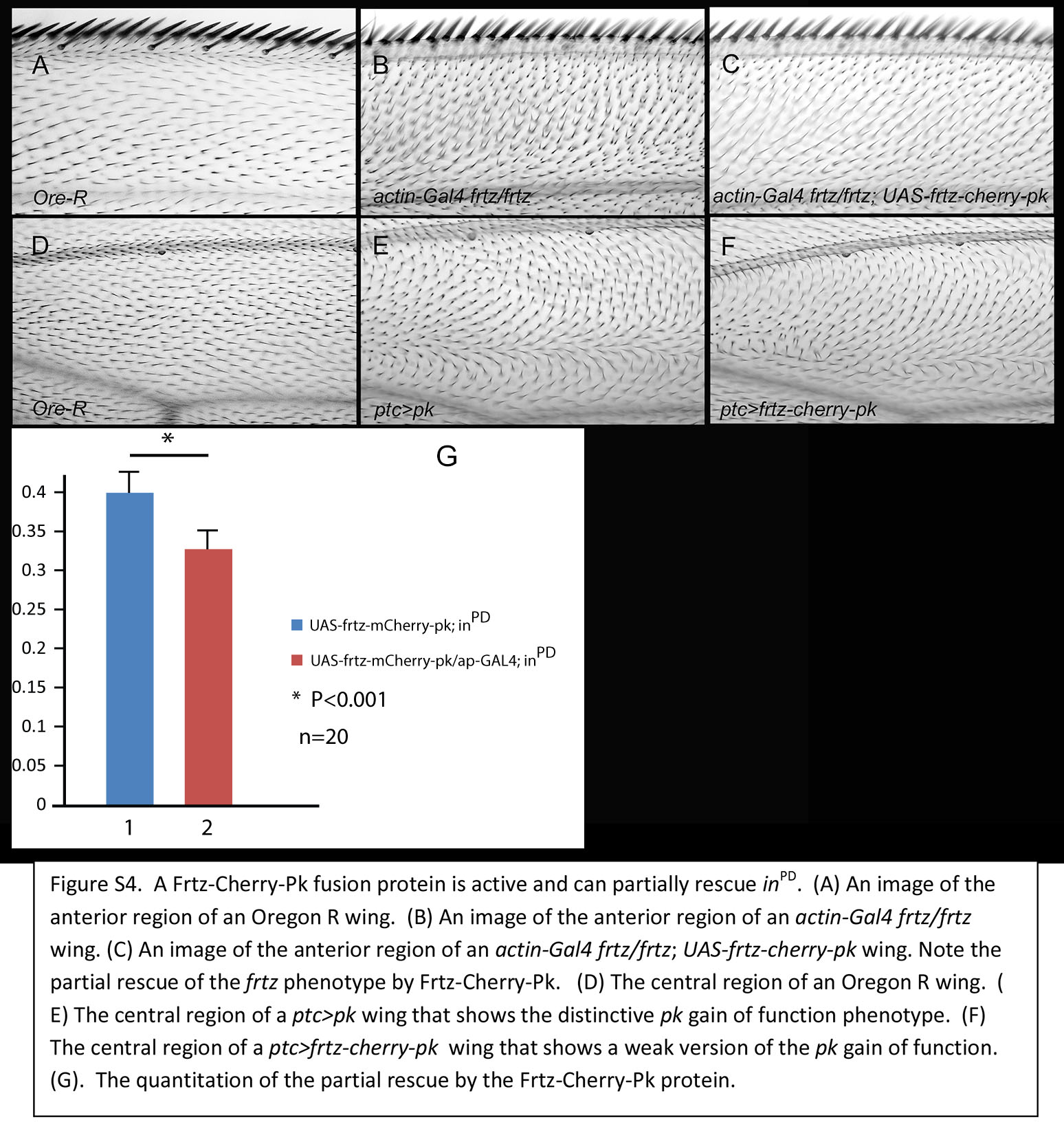

Supplement: Supplementary file 4 [file 1323FigureS4.jpg]

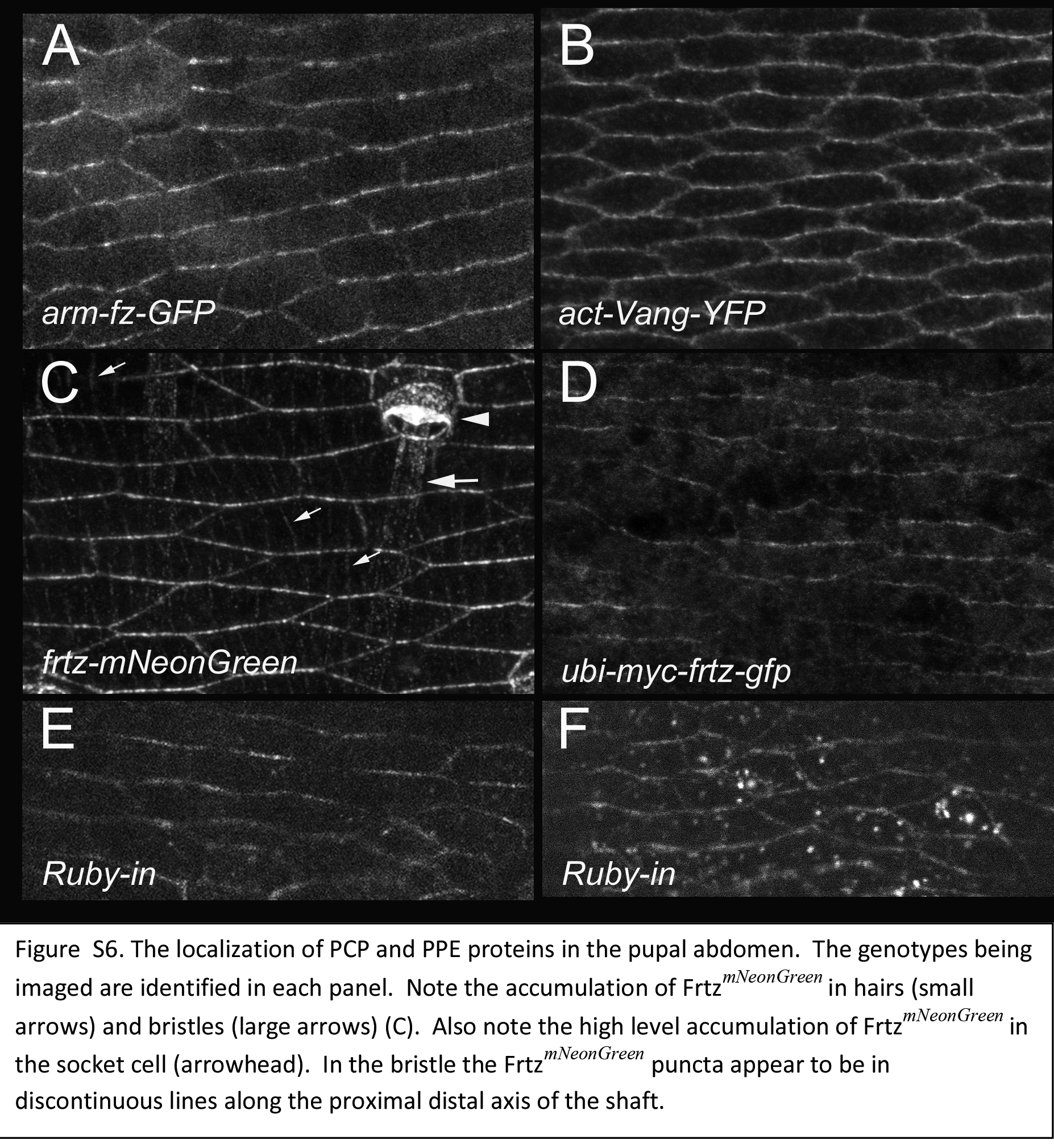

Supplement: Supplementary file 6 [file 1323FigureS6.tif]
