# Supplementary material for: Planar Cell Polarity Effector Fritz Interacts with Dishevelled and Has Multiple Functions in Regulating PCP
Source: G3 (Bethesda). 2017 Mar 2;7(4):1323–37. doi: 10.1534/g3.116.038695 (PMC5386880; doi:10.1534/g3.116.038695)
Supplement: Supplementary file 2 [file 1323FigureS2.pdf]

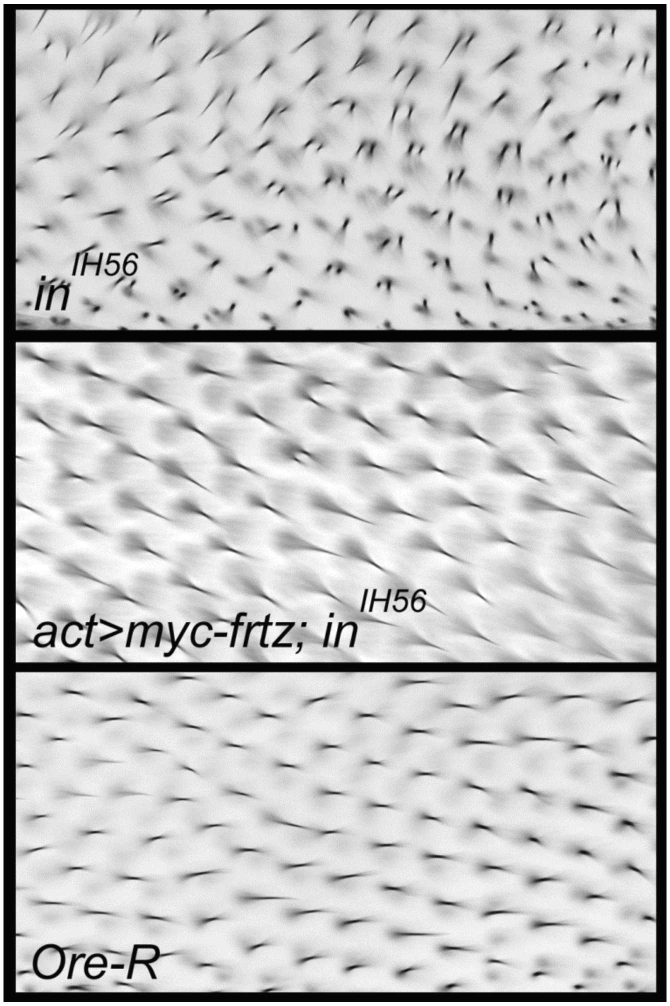

Figure S2. Suppression of a strong *in* allele by the mild overexpression of *myc-frtz*. All panels show the same region of the wing. The upper panel shows the central region of an *in*^{IH56} wing. Note the strong multiple hair cell and abnormal hair polarity phenotypes. The middle panel shows the suppressed phenotype of *act>myc-frtz; in*^{IH56} wings. Note the much weaker multiple hair and polarity phenotypes. The lower panel shows a wild type Oregon-R wing.
