# Supplementary material for: Planar Cell Polarity Effector Fritz Interacts with Dishevelled and Has Multiple Functions in Regulating PCP
Source: G3 (Bethesda). 2017 Mar 2;7(4):1323–37. doi: 10.1534/g3.116.038695 (PMC5386880; doi:10.1534/g3.116.038695)
Supplement: Supplementary file 3 [file 1323FigureS3.pdf]

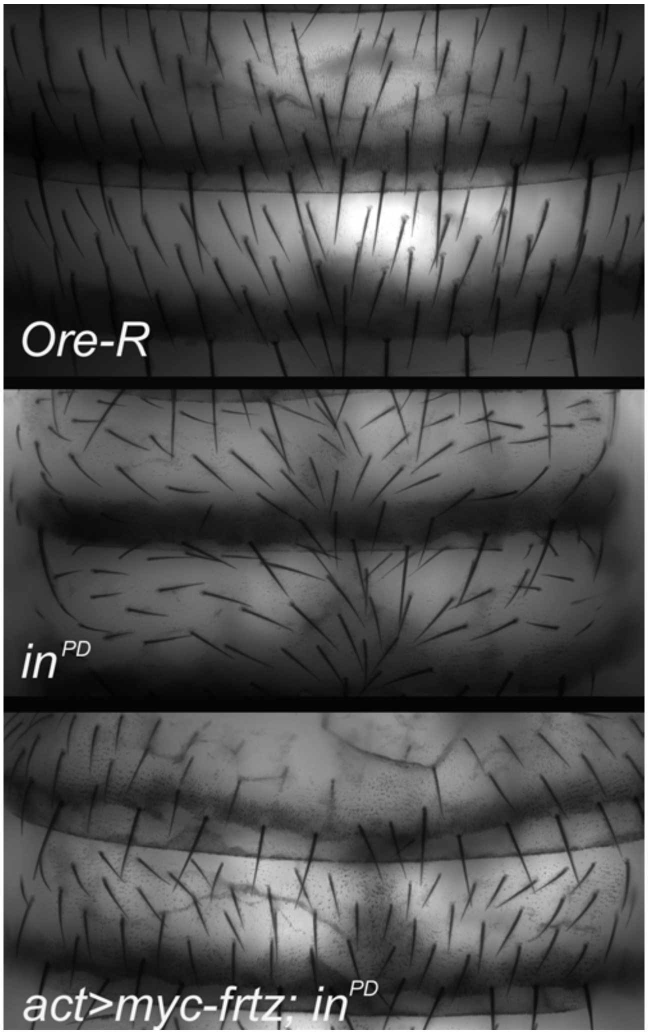

Figure S3. The abdomen of Ore-R (wild type), *in*^{PD} and *act>myc-frtz; in*^{PD}. Note the strong effect of *in*^{PD} on bristle polarity and the substantial rescue by the expression of *myc-frtz.*
