# Supplementary material for: Planar Cell Polarity Effector Fritz Interacts with Dishevelled and Has Multiple Functions in Regulating PCP
Source: G3 (Bethesda). 2017 Mar 2;7(4):1323–37. doi: 10.1534/g3.116.038695 (PMC5386880; doi:10.1534/g3.116.038695)
Supplement: Supplementary file 5 [file 1323FigureS5.pdf]

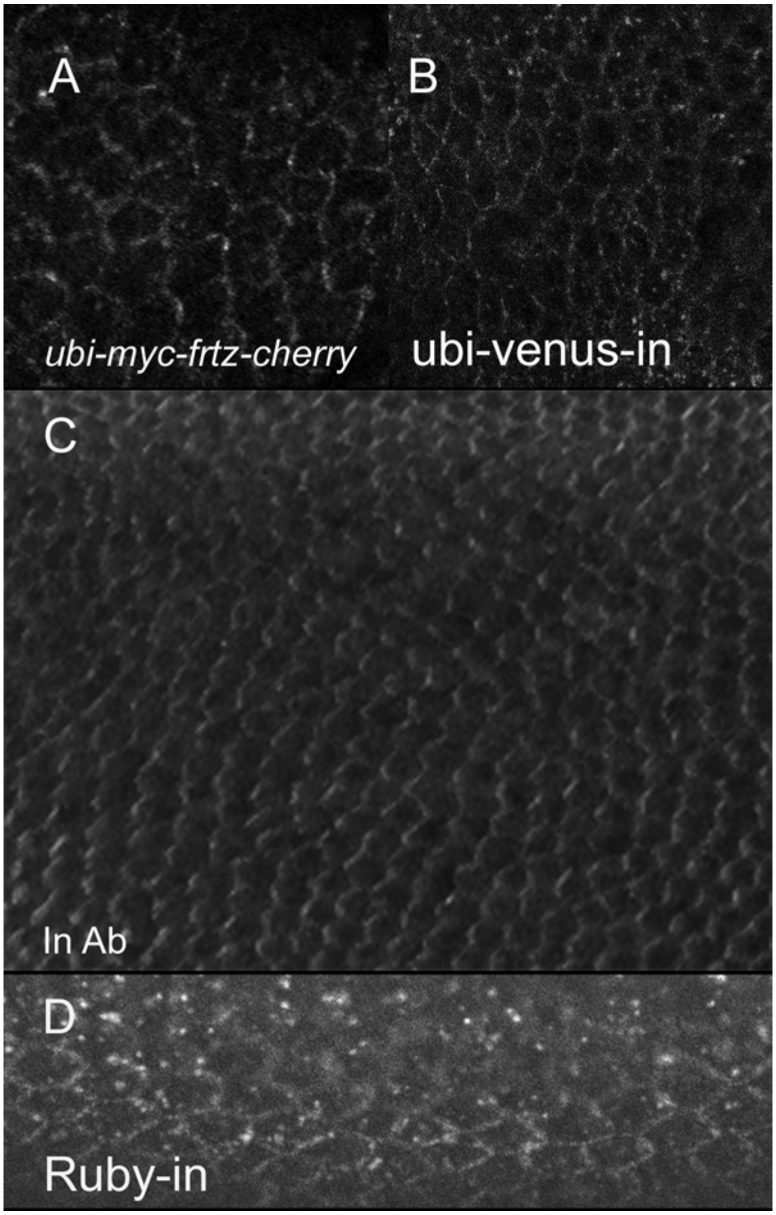

Figure S5. Localization of PPE proteins. (A) The accumulation of transgenic Myc-Frtz-Cherry. (B) The accumulation of Venus-In. (C) Immunostaining of the endogenous In protein. (D) An image of Ruby-In in a living pupal wing. Note the many puncta in B and D vs C.
