# Supplementary material for: Planar Cell Polarity Effector Fritz Interacts with Dishevelled and Has Multiple Functions in Regulating PCP
Source: G3 (Bethesda). 2017 Mar 2;7(4):1323–37. doi: 10.1534/g3.116.038695 (PMC5386880; doi:10.1534/g3.116.038695)
Supplement: Supplementary file 7 [file 1323FigureS7.pdf]

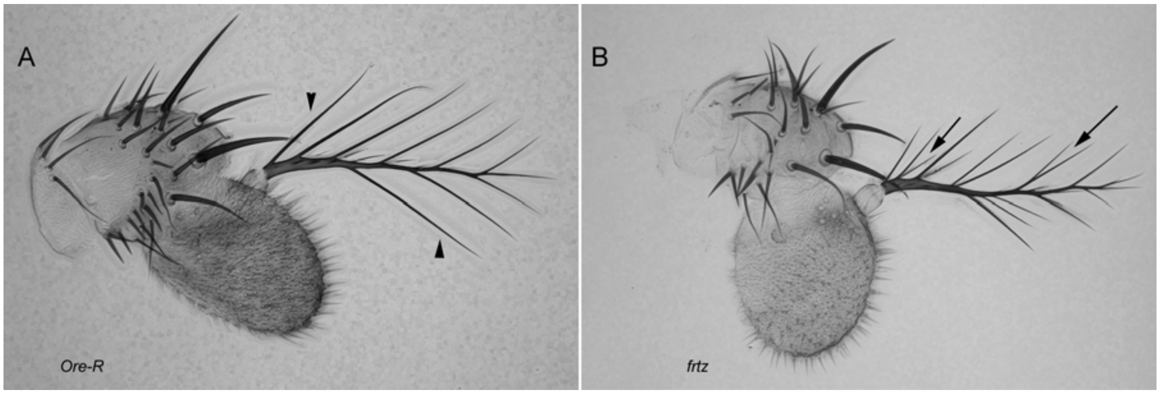

Figure S7. *frtz* function is needed for the normal development of the arista. (A) The arista (terminal segment of the antenna) in Oregon-R contains long unbranched laterals (arrowheads). (B). The arista (terminal segment of the antenna) in *frtz*^{30} contains branched laterals that are shorter than normal (arrows).
