# Supplementary material for: Planar Cell Polarity Effector Fritz Interacts with Dishevelled and Has Multiple Functions in Regulating PCP
Source: G3 (Bethesda). 2017 Mar 2;7(4):1323–37. doi: 10.1534/g3.116.038695 (PMC5386880; doi:10.1534/g3.116.038695)
Supplement: Supplementary file 8 [file 1323FigureS8.pdf]

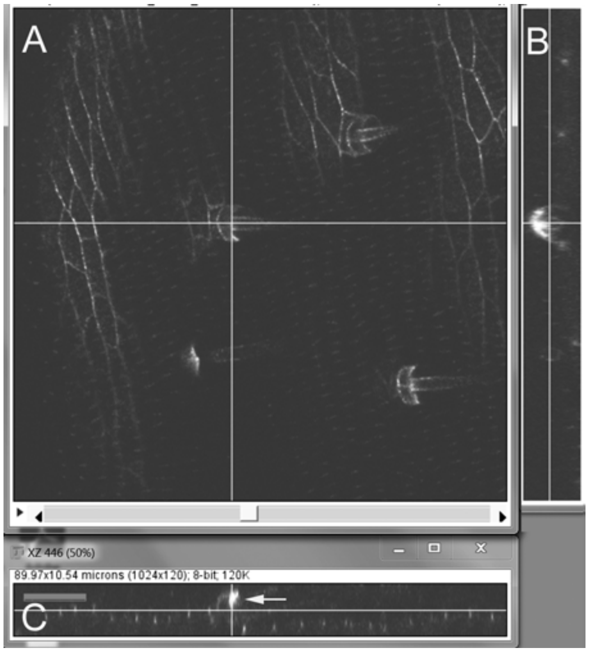

Figure S8. Orthogonal view of Frtz-NG in the pupal abdomen. (A) An optical section from a living $frtz^{mNeonGreen}$ pupal abdomen. The crossing lines indicate the planes for the orthogonal views. (B and C) Orthogonal sections of A. The arrow in C points to the location in the socket cell with the highest concentration of $Frtz^{mNeonGreen}$. $Frtz^{mNeonGreen}$ can be seen in the hairs and bristles.
