# Supplementary material for: Planar Cell Polarity Effector Fritz Interacts with Dishevelled and Has Multiple Functions in Regulating PCP
Source: G3 (Bethesda). 2017 Mar 2;7(4):1323–37. doi: 10.1534/g3.116.038695 (PMC5386880; doi:10.1534/g3.116.038695)
Supplement: Supplementary file 9 [file 1323FileS1.docx]

**Supplementary methods**

**Plasmid construction**

***frtz^mNeonGreen^* CRISPR/gRNA plasmid construction :**

gRNA primers:

PCFD4 FRTZ 5: TATATAGGAAAGATATCCGGGTGAACTTCGCCGCCCATCCGACGACGCGGTTTTAGAGCTAGAAATAGCAAG

PCFD4 FRTZ 3: ATTTTAACTTGCTATTTCTAGCTCTAAAACCACAACGGTTAAGCGCTGCCGACGTTAAATTGAAAATAGGTC

The above primers amplified a 600bp fragment by PCR which includes both 5gRNA and 3gRNA. The fragment was inserted into BbsI digested *pCFD4-U6:1_U6:3* tandem gRNAs vector (Addgene plasmid # 49411) and ligated by T4 DNA ligase.

***frtz^mNeonGreen^* CRISPR HDR template** *pHD-DsRed-frtz-mNeonGreen*

5’ flanking region primers

5+7th exon 5': CCGGAATTCTCGGCGGTGACCAGCGACGA (EcoRI)

5+7th exon 3': ATAAGAATGCGGCCGCGACCACGCCGAAGTGGACCA (NotI)

mNeonGreen primers:

mNeonGreen 5': ATAAGAATGCGGCCGCATGGTGAGCAAGGGCGAGGA (NotI)

mNeonGreen 3': CTACATATGTTACTTGTACAGCTCGTCCA (NdeI)

3’ flanking region primers

3 arm 5': GGAAGATCTAGGCGTACACCACACAACGG (BglII)

3 arm 3': CCGCTCGAGCGCCGGGCCAAGTAGCAAGG (XhoI)

Both 5’ and 3’ flanking regions were amplified by PCR using genomic DNA. mNeonGreen clone was purchased from Allele Biotech and amplified by PCR. The fragments were digested with corresponding restriction enzymes and ligated into the same restriction enzymes digested *pHD-DsRed* (Addgene plasmid #51434) vector. The 3 nucleotides PAM sequence following the gRNA on the HDR template were mutated so the Cas9 nuclease would not recut edited DNA.

***in^PD^* CRISPR/Cas9 gRNA plasmid:**

We designed two guide RNAs (gRNA) specifically targeting the 5’ and 3’ ends of the in gene and subcloned these into the pU6-3-gRNA plasmid behind the snRNA:U6-3 promoter (Addgene plasmid #45946).

5 gRNA 5’: CTTCGGGAAACTCAAAACCCACCG

5 gRNA 3’: AAACCGGTGGGTTTTGAGTTTCCC

3 gRNA 5' : CTTCGTCTTTTGACTTCTGGTGCG

3 gRNA 3' : AAACCGCACCAGAAGTCAAAAGAC

Oligos were phosphorylated using T4 PNK. Then complementary primers were self-annealed by running the following thermocycler program: 95°C for 5 min, then ramp to 25°C at a rate of -5°C/min. The *pU6-BbsI-chiRNA* (Addgene plasmid #45946) plasmid was digested with BbsI enzyme and de-phosphorylated. The oliogs were mixed with cut *pU6-BbsI-chiRNA* plasmid, the mix ligated and transformed into E. coli competent cells.

***in^PD^* CRISPR/Cas9 HDR template *pHD-DsRed-In^PD^***

Primers for 5’ and 3’ *inturned* flanking region

In5 flank 5 – CCGGAATTCCAGCAGACAACTGTTCCCAT (EcoRI)

In5 flank 3 – CTAGCTAGCAAGCCGAGATCCGATCTCAG (NheI)

In3 flank 5 – AACTGCAGAACCAATGCATTGGAAACATCGTGCGAATGTGTT (PstI)

In3 flank 3 – CCGCTCGAGCGGAGCACATGGTTTTGGTCTCT (XhoI)

Both 5’ and 3’ flanking regions (about 1.5kb each) were cloned by PCR using genomic DNA and digested with corresponding restriction enzyme. *pHD-DsRed* (Addgene plasmid #51434) was digested with the same restriction enzymes and ligated with both flanking regions.

***mRuby-In* CRISPR HDR template *pHD-DsRed-mRuby-In***

The following primers were used to amplify the In CDS and 1.5kb 3’ flanking region including 3’ UTR from *in* genomic clone, then ligated into XbaI digested *pc-DNA3-mRuby* vector (addgene plasmid # 40260) using Gibson Assembly to make *pc-DNA3-mRuby-In CDS-In3*

5':GGGATGGACGAGCTGTACAAGGAAGAACAAAAGCTAATCTCAGAAATGCGCAAATCGCCGGCCAGC

3': TATAGAATAGGGCCCTCTAGAGAATTGTGTTCCTCTTGAAATACC

Using the flowing primers to amplify 1.5kb In 5’ flanking region including 5’ UTR. The PCR product was digested using the specific restriction enzyme and ligated into same restriction enzymes digested *pHD-DsRed* vector (Addgene plasmid #51434) to make *pHD-DsRed-In5* vector

5' primer: ccggaattcCAGCAGACAACTGTTCCCAT (EcoRI)

3' primer: ctagctagcAGTTTACCGGGGATAGTTAAC (NheI)

The following primer were used to amplify the mRuby2-In CDS-In 3. The PCR product was ligated into *pHD-DsRed-In5* vector (digested with NotI) using Gibson Assembly.

5' primer: TATCCCCGGTAAACTGCTAGCATGGTGTCTAAGGGCGAAGAG

3' primer: GTGTGCATATGTCCGCGGCCGTGTTCCTCTTGAAATACCTTC

The 3 nucleotides PAM sequence following the gRNA on the HDR template were mutated so the Cas9 nuclease would not recut edited DNA.

***pWUM6- frtz-mCherry* plasmid construction**

*pWUM6* vector was digested with EcoRI and XbaI restriction enzymes and the fragments were amplified using the following primers and assembled into the digested vector using Gibson Assembly.

Primers for amplifying *frtz* fragment
frtz5': CGGCGTAGATCTGGTACCTATGCTGCTCAGCGAGACCCATTT
frtz3': CTCCTCGCCCTTGCTCACCATGCGATCGCTGACCACGCCGAAGTGGACCA

Primers for amplifying *mCherry* fragment

mcherry5': TGGTCCACTTCGGCGTGGTCAGCGATCGCATGGTGAGCAAGGGCGAGGAG
mcherry3': CGGCATGGACGAGCTGTACAAGTAACTAGGCCGGCCTTCAAGGCCT

***pWUM6 -Venus-in* construct**

*pWUM6* vector was digested with corresponding restriction enzymes and the fragments were amplified using the following primers and ligated into the digested vector.

Primers for amplifying Venus fragment

Venus 5': CTAGTCTAGAATGGTGAGCAAGGGCGAGGAG  (XbaI)
Venus3' : ATAAGAATGCGGCCGCCGTGGACCGGTGCTTGTACAGCTC (NotI)

Primers for amplifying In fragment

In 5: ATAAGAATGCGGCCGCATGCGCAAATCGCCGGCCAG (NotI)
In 3: CGACGCGTTTATCCCATTGAGAAGAAGGACA (MluI)

To get truncated forms of fritz cDNA, we used the following primers and the PCR products were sub-cloned into *pGBKT7* vector using NdeI and EcoRI restriction sites.

1) Primers for *pGBKT7-frtz* (1-400aa)

5′ frtz-hybrid: GGGAATTCCATATGCTGCTCAGCGAGACC;

DBD-frtzN-3’: CCGGAATTCTTAGTGCGACAGATCCAGCAAG

2) Primers for *pGBKT7-frtz* (401 aa-end)

DBD-frtzC-5’: GGGAATTCCATATGTACTTCGTGGCCCAGCCA

3′fritz-hybrid: CCGGAATTCTTATTAGACCACGCCGAAGTGGA.

3) Primers for *pGBKT7-frtz* (201-400aa)

Dfrtz400-5’: GGGAATTCCATATGAACGCCAGCTTCGATC

DBD-frtzN-3’: CCGGAATTCTTAGTGCGACAGATCCAGCAAG

4) Primers for *pGBKT7-frtz* (WD40:308aa-385aa)

DBD-Fritz-WD5: GGAATTCCATATGCAGATCTGCTCCTTTGCCTTC

DBD-Fritz-WD3: CGGAATTCTTACAAGCTGATGGCCAATGGTG

5) Primers for *pGBKT7-frtz* (100-700aa)

300 primer 5 – GGAATTCCATATGCTCTTCTCACACGGCCTGAT

2100 primer 3 - CCGGAATTCCTCGCCAGCGAAGATGTTGG

6) Primers for *pGBKT7-frtz* (200-600aa)

600 primer 5 – GGAATTCCATATGAACGCCAGCTTCGATCTGTT

1800 primer 3 - CCGGAATTCTCGTGGATTATGGCAGCTGC

7) Primers for *pGBKT7-frtz* (300-500aa)

900 primer 5 – GGAATTCCATATGACCTCCATTCCAATGGGCGC

1500 primer 3 - CCGGAATTCACATAGTTGGCTATCTTGTG

**Sub-cloning for yeast-two-hybrid assays**

Full-length in cDNA was sub-cloned into *pGADT7* vector from NdeI-BamHI. The following primers were used:

Inturn-th5: TCTAGGGAATTTCCATATGCGCAAATCGCCGGCCAG;

Inturn-th3: GATCGCGGATCCATGTCATCCCATTGAGAAGAAGGA.

Full-length fy cDNA was sub-cloned into *pGADT7* vector from NcoI-BamHI. The following primers were used:

pGBKT7-5′: CATGCCATGGAGATGTCCATCTATTTGTTATG;

pGBKT7-3′: CGCGGATCCTTATCACCAACATACTGACTTC;

Full-length of fritz cDNA was sub-cloned into *pGBKT7* vector from NdeI-EcoRI . The following primers were used:

5′ frtz-hybrid: GGGAATTCCATATGCTGCTCAGCGAGACC;

3′fritz-hybrid: CCGGAATTCTTATTAGACCACGCCGAAGTGGA.

Full-length *dsh* cDNA was sub-cloned into both *pGBKT7* and *pGADT7* vectors from NdeI-BamHI. The following primers were used:

AD/Bk-Dsh5':CATATGGACGCGGACAGGGGCGG

AD/Bk-Dsh 3': GGATCCCTACAATACGTAATTAAATACGG

Full-length of intu cDNA was sub-cloned into both *pGBKT7* and *pGADT7* vectors from EcoRI-BamHI. The following primers were used:

Intu 5': CCGGAATTCATGGCCTCTGTGGCTTCGTGC (EcoRI)

Intu 3': CGCGGATCCCTACAAGGTTAACCCAAAGAA (BamHI)

Full-length of wdpcp cDNA was sub-cloned into both *pGBKT7* and *pGADT7* from NdeI-EcoRI. The following primers were used:

WDPCP 5' GGAATTCCATATGATGTTTTCGTCTCTACATTCAGC (NdeI)

WDPCP 3' CCGGAATTCTTACACCAGACCAAAGTGAATCAT (EcoRI)

Full-length of dvl2 cDNA was sub-cloned into both *pGBKT7* and *pGADT7* vectors from NdeI-XhoI. The following primers were used:

dvl2 5' GGAATTCCATATGATGGCGGGTAGCAGCACTGG (ndeI)

dvl2 3' CCGCTCGAGCTACATAACATCCACAAAGAACTC (XhoI)

**Frtz fragment deletion assay constructs**

Each fragment deletion construct carried a 100aa fragment deletion (except the last one which has 150aa deletion) of the full length *frtz* cDNA. In addition, there is a 26 aa spacer between the two *frtz* fragments, including a *myc* tag and several random aa.

1) *pGBKT7-frtz* (100-951aa). Cut *pGBKT7* vector with NdeI and Xmal, and perform the Gibson assembly. The following primers were used to amplify the fragments by PCR.

frtz 100-951 5' ATCTCAGAGGAGGACCTGCATATGTTCTCACACGGCCTGATC

frtz 100-951 3' GGTCGACGGATCCCCGGGTTATTAGACCACGCCGAAGTGGAC

2) *pGBKT7-frtz* (1-100aa & 201-951aa)*.* Cut *pGBKT7* vector with XhoI and Xmal, and perform the Gibson assembly. The following primers were used to amplify the fragments by PCR.

Primers for amplifying 1-100aa fragment:

frtz 1-100 200-951 1st 5' CAGCTATTTCTACTGATTTTTCCTCGAGAAGACCTTGACATG

frtz 1-100 200-951 1st 3' CAGGTCTTCTTCTGAGATTAGCTTTTGTTCTTCGCCGTGTGA GAAGAGGAGCAG

Primers for amplifying 201-951aa fragment

frtz 1-100 200-951 2nd 5' GAAGAACAAAAGCTAATCTCAGAAGAAGACCTGCGCCACC TCACAGTTAACGCC

frtz 1-100 200-951 2nd 3' GCCGCTGCAGGTCGACGGATCCCCGGGTTATTAGACCACGC CGAAGTGGAC

3) *pGBKT7-frtz* (1-200aa & 301-951aa)*.* Cut *pGBKT7* vector with XhoI and Xmal, and perform the Gibson assembly. The following primers were used to amplify the fragments by PCR

Primers for amplifying 1-200aa fragment

frtz 1-200 300-951 1st 5' (same with frtz 1-100 200-951 1st 5' ) CAGCTATTTCTACTGATTT TTCCTCGAGAAGACCTTGACATG

frtz 1-200 300-951 1st (same with frtz 1-100 200-951 2nd 3') 3'CAGGTCTTCTTCTGAGATT AGCTTTTGTTCTTCATCGAAGCTGGCGTTAACTGT

Primers for amplifying 301-951 fragment

frtz 1-200 300-951 2nd 5'GAAGAACAAAAGCTAATCTCAGAAGAAGACCTGCAGCGCA CCGCCATTACCTCC

frtz 1-200 300-951 2nd (same with frtz 1-100 200-951 2nd 3') GCCGCTGCAGGTCGACGG ATCCCCGGGTTATTAGACCACGCCGAAGTGGAC

4) *pGBKT7-frtz* (1-300aa & 401-951aa). Cut *pGBKT7* vector with XhoI and Xmal, and perform the Gibson assembly. The following primers were used to amplify the fragments by PCR

Primers for amplifying 1-300aa fragment

frtz 1-300 400-951 1st 5': (same with frtz 1-100 200-951 1st 5' ) CAGCTATTTCTACTGATTT TTCCTCGAGAAGACCTTGACATG

frtz 1-300 400-951 1st 3': CAGGTCTTCTTCTGAGATTAGCTTTTGTTCTTCCATTGGAAT GGAGGTAATGGC

Primers for amplifying 401-951 fragment

frtz 1-300 400-951 2nd 5': GAAGAACAAAAGCTAATCTCAGAAGAAGACCTGCTGGATC TGTCGCACTACTTC

frtz 1-300 400-951 2nd 3': (same with frtz 1-100 200-951 2nd 3') GCCGCTGCAGGTCGACG GATCCCCGGGTTATTAGACCACGCCGAAGTGGAC

5) *pGBKT7-frtz* (1-400aa & 501-951aa)*.* Cut *pGBKT7* vector with XhoI and Xmal, and perform the Gibson assembly. The following primers were used to amplify the fragments by PCR

Primers for amplifying 1-400aa fragment

frtz 1-400 500-951 1st 5' : (same with frtz 1-100 200-951 1st 5' ) CAGCTATTTCTACTGATTT TTCCTCGAGAAGACCTTGACATG

frtz 1-400 500-951 1st 3' : CAGGTCTTCTTCTGAGATTAGCTTTTGTTCTTCCTGGGCCA CGAAGTAGTGCGA

Primers for amplifying 501-951 fragment

frtz 1-400 500-951 2nd 5': GAAGAACAAAAGCTAATCTCAGAAGAAGACCTGTGCCTG ATTACGCTGCACAAG

frtz 1-400 500-951 2nd 3': (same with frtz 1-100 200-951 2nd 3') GCCGCTGCAGGTCGACG GATCCCCGGGTTATTAGACCACGCCGAAGTGGAC

6) *pGBKT7-frtz* (1-500aa & 601-951aa). Cut *pGBKT7* vector with XhoI and Xmal, and perform the Gibson assembly. The following primers were used to amplify the fragments by PCR

Primers for amplifying 1-500aa fragment

frtz 1-500 600-951 1st 5': (same with frtz 1-100 200-951 1st 5' ) CAGCTATTTCTACTGATTT TTCCTCGAGAAGACCTTGACATG

frtz 1-500 600-951 1st 3': CAGGTCTTCTTCTGAGATTAGCTTTTGTTCTTCGTTGGCTAT CTTGTGCAGCGT

Primers for amplifying 601-951aa fragment

frtz 1-500 600-951 2nd 5': GAAGAACAAAAGCTAATCTCAGAAGAAGACCTGGTGGCCT TCAGTCAGGCAGCT

frtz 1-500 600-951 2nd 3': (same with frtz 1-100 200-951 2nd 3') GCCGCTGCAGGTCGACG G ATCCCCGGGTTATTAGACCACGCCGAAGTGGAC

7) *pGBKT7-frtz* (1-600aa &701-951aa)*.* Cut *pGBKT7* vector with XhoI and Xmal, and perform the Gibson assembly. The following primers were used to amplify the fragments by PCR

Primers for amplifying 1-600aa fragment

frtz 1-600 700-951 1st 5': (same with frtz 1-100 200-951 1st 5' ) CAGCTATTTCTACTGATTT TTCCTCGAGAAGACCTTGACATG

frtz 1-600 700-951 1st 3': CAGGTCTTCTTCTGAGATTAGCTTTTGTTCTTCGATTATGGC AGCTGCCTGACT

Primers for amplifying 700-951aa fragment

frtz 1-600 700-951 2nd 5': GAAGAACAAAAGCTAATCTCAGAA GAAGACCTGGTTTCA ATAGCGCCGCCAACA

frtz 1-600 700-951 2nd 3': (same with frtz 1-100 200-951 2nd 3') GCCGCTGCAGGTCGACG GATCCCCGGGTTATTAGACCACGCCGAAGTGGAC

8) *pGBKT7-frtz* (1-700aa &801-951aa)*.* Cut *pGBKT7* vector with XhoI and Xmal, and perform the Gibson assembly. The following primers were used to amplify the fragments by PCR

Primers for amplifying 1-700aa fragment

frtz 1-700 800-951 1st 5': (same with frtz 1-100 200-951 1st 5' ) CAGCTATTTCTACTGATTT TTCCTCGAGAAGACCTTGACATG

frtz 1-700 800-951 1st 3': CAGGTCTTCTTCTGAGATTAGCTTTTGTTCTTCCAGCGAAGA TGTTGGCGGCGC

Primers for amplifying 801-951aa fragment

frtz 1-700 800-951 2nd 5': GAAGAACAAAAGCTAATCTCAGAAGAAGACCTGGCCAT GCTGCCATCGCTCACC

frtz 1-700 800-951 2nd 3': (same with frtz 1-100 200-951 2nd 3') GCCGCTGCAGGTCG ACGGATCCCCGGGTTATTAGACCACGCCGAAGTGGAC

9) *pGBKT7-frtz* (1-800aa). Cut *pGBKT7* vector with XhoI and Xmal, and perform the Gibson assembly. The following primers were used to amplify the fragments by PCR.

frtz 1-800 5': (same with frtz 1-100 200-951 1st 5' )

CAGCTATTTCTACTGATTTTTCCTCGAGAAGACCTTGACATG

frtz 1-800 3': GCAGGTCGACGGATCCCCGGGTTATTACGATGGCAGCATGGCGGG
