# Supplementary material for: Planar Cell Polarity Effector Fritz Interacts with Dishevelled and Has Multiple Functions in Regulating PCP
Source: G3 (Bethesda). 2017 Mar 2;7(4):1323–37. doi: 10.1534/g3.116.038695 (PMC5386880; doi:10.1534/g3.116.038695)
Supplement: Supplementary file 11 [file 1323TableS1.pdf]

Table S1

Analysis of Frtz WD40 motifs

A

Drosophila_Frtz is a WD40 protein. The average score is : 53.17

The esstimated Q3 accuracy is : 86$\pm$5%

Topology of Drosophila_Frtz

Repeats	Score	Start — End	Strand _d	Loop _da	Strand _a	Loop_ab	Strand _b	Loop_bc	Strand _c	Loop _cd	Hotspots on the top face
WD1	52.07	123-167	VFEKYL	VGKLASEV	ITDAFF	TRS	HIVLAY	NTN	QLTVVH	LQRPNAR	NA
WD2	20.55	168-242	SQGPEK	IANMDPR	IFHVII	PGATERKLSRHLTVNASFD	LFVVWT	QSSQNEVYPWRPTIRDQDRA	NIHVFK	IKGLQ	R180 F182
WD3	33.51	243-295	LESIAY	CWSEND	PLCVDF	LRSSES	QIITLE	QKVSRKGDISA	EICSYE	LAAGKM	D254 L256
WD4	71.81	296-339	QRTAIT	SIPMGAQ	ICSFAF	SPDQE	KLFLGS	VDR	NICLHD	LVQQS	Q308
WD5	51.88	340-382	TKYANQ	IEIV	PNQCAW	HCDSA	MLCVAN	ERS	VLQCFD	LALATIG	N351
WD6	40.12	383-423	HQLVSE	NVTPSS	LLDLSH	YFVAQP	TLLSVA	FSRKP	DLSTFK		L396

B

Human_Frtz is a WD40 protein. The average score is : 46.05

The esstimated Q3 accuracy is : 85$\pm$5%

Topology of Human_Frtz

Repeats	Score	Start — End	Strand _d	Loop _da	Strand _a	Loop_ab	Strand _b	Loop _bc	Strand _c	Loop_cd	Hotspots on the top face
WD1	53.61	115-153	RCVLSK	WKNKY	VCQLLF	GSG	VLVSLS	LSGP	QLEKW	IDR	Y125
WD2	40.79	154-196	SLVGKL	ISDT	ISDALL	TDSF	IILSFL	AQN	KLCFIQ	FTKKMESS	NA
WD3	23.39	197-252	DVNKRL	EKLSALD	YKIFYY	EIPGPINKTTER	HLAINC	VHDR	VVCWWP	LVNDDAWPW	D209 K211
WD4	30.96	253-326	APISSE	KDRAN	LLLLGY	AQGRL	EVLSSV	RTEWDPLDVRFGTKQPY	QVFTVE	HSVSVDKEPMADSCIYECIRNKI	N263 L265
WD5	69.04	327-370	QCVSVT	RIPLKSKA	ISCCRN	VTED	KLILGC	EDS	SLILYE	THRRV	E357
WD6	60.22	371-424	TLLAQT	ELLPS	LISCHP	SGA	ILLVGS	NQG	ELQIFD	MALSPINIQLLAEDRLPRE	I383
WD7	35.67	425-477	TLQFSK	LFDASSS	LVQMQW	IAPQVVSQKGEGSDIYD	LLFLRF	ERGPL	GVLLFK		NA

The Drosophila Frtz and human WDPCP proteins were analyzed using the WD40-repeat protein Structure Predictor (WDSP) program. Predicted hot spots for contacting other proteins are shown in red. The locations of the various WD40 loops and strands are indicated.
