# Supplementary material for: Planar Cell Polarity Effector Fritz Interacts with Dishevelled and Has Multiple Functions in Regulating PCP
Source: G3 (Bethesda). 2017 Mar 2;7(4):1323–37. doi: 10.1534/g3.116.038695 (PMC5386880; doi:10.1534/g3.116.038695)
Supplement: Supplementary file 12 [file 1323TableS2.pdf]

Table S2
Sites altered in mutagenesis experiments.

Candidates	Hotspot (predicted by WDSP)	Conserved in Frtz WD40	Conserved in other WD40 proteins	On protein surface
Q308	Yes	No	No	Yes
C310	No	No	No	Yes
V326	No	No	Yes	Yes
T340	No	Yes	No	Yes
A343	No	Yes	No	Yes
Q345	No	Yes	No	Yes
E347	No	Yes	No	Yes
N351	Yes	No	Yes	Yes
N366	No	No	No	Yes
E367	No	No	Yes	Yes
A377	No	Yes	No	Yes
Q384	No	Yes	No	Yes

The yeast two hybrid system was used to determine the consequences of mutations shown on the ability of the Frtz protein to interact with Inturned. Mutating of these sites in one protein did not block the ability of Frtz and In to interact.
