# Supplementary material for: Planar Cell Polarity Effector Fritz Interacts with Dishevelled and Has Multiple Functions in Regulating PCP
Source: G3 (Bethesda). 2017 Mar 2;7(4):1323–37. doi: 10.1534/g3.116.038695 (PMC5386880; doi:10.1534/g3.116.038695)
Supplement: Supplementary file 13 [file 1323TableS3.pdf]

Table S3

Protein Interactions detected in this paper and Wang et al; 2014.

Source	Protein 1	Protein 2	Yeast 2-hybrid result
human	Intu	Dvl2	+
human	Intu	WDPCP	+
human	WDPCP	DVl2	-
Drosophila	In	Dsh	-
Drosophila	In	Frtz	+
Drosophila	Frtz	Dsh	+
Drosophila	Fy	Dsh	+
Drosophila	In	Fy	+
Drosophila	Frtz	Fy	-
Drosophila	In	Frtz	+
