# Supplementary material for: Planar Cell Polarity Effector Fritz Interacts with Dishevelled and Has Multiple Functions in Regulating PCP
Source: G3 (Bethesda). 2017 Mar 2;7(4):1323–37. doi: 10.1534/g3.116.038695 (PMC5386880; doi:10.1534/g3.116.038695)
Supplement: Supplementary file 14 [file 1323TableS4.pdf]

Table S4 Landing sites test for *in*, *fy* and *frtz* transgenic proteins expression

Genotype	Landing site	Rescue or not	Zig-zag expression
pWUM6-in-venus	*attp1*	Yes	No
	vk00027	Yes	No
	attp 2	Yes	No
pWUM6-venus-in	*attp1*	Yes	Yes
pWUM6-fy-mcerulean3	*attp1*	Yes	No
	vk00027	Yes	No
pWUM6-frtz-mcherry	*attp1*	Yes	Yes
	vk00027	Yes	No
